# Supplementary material for: Observation of first- and second-order dissipative phase transitions in a two-photon driven Kerr resonator
Source: Nat Commun. 2025 Mar 10;16:1954. doi: 10.1038/s41467-025-56830-w (PMC11893805; doi:10.1038/s41467-025-56830-w)
Supplement: Supplementary file 1 — Supplementary information [file 41467_2025_56830_MOESM1_ESM.pdf]

Supplementary information for

Observation of first- and second-order dissipative phase transitions in a two-photon driven Kerr resonator

CONTENTS

Supplementary Note 1. Supplementary Figures 2

Supplementary Note 2. Setup and device 6
A. Device design 6
B. Device fabrication 6
C. Experimental setup 7
D. Characterization of the device parameters 7
E. Calibration of Input Attenuation, Amplifier Noise, and Gain 9
F. Moments reconstruction and squeezing parameter 10

Supplementary Note 3. Measurement protocols 12

Supplementary Note 4. Modeling of the system 15
A. Hamiltonian parameters 15
B. Open-system parameters 19

Supplementary Note 5. Parameter estimation 20
A. Testing the validity of the model 21
B. Determination of the final parameter set 22

Supplementary Note 6. Theory of dissipative phase transitions 22
A. Open-system dynamics and Quantum Trajectories 22
1. Ergodicity 22
B. The Liouvillian spectrum and phase transitions 22
1. Second order phase transition 23
2. First-order dissipative phase transition and the Liouvillian spectrum 23
C. Extracting the Liouvillian gap from symmetry breaking trajectories 24

Supplementary References 26

Supplementary Note 1. SUPPLEMENTARY FIGURES

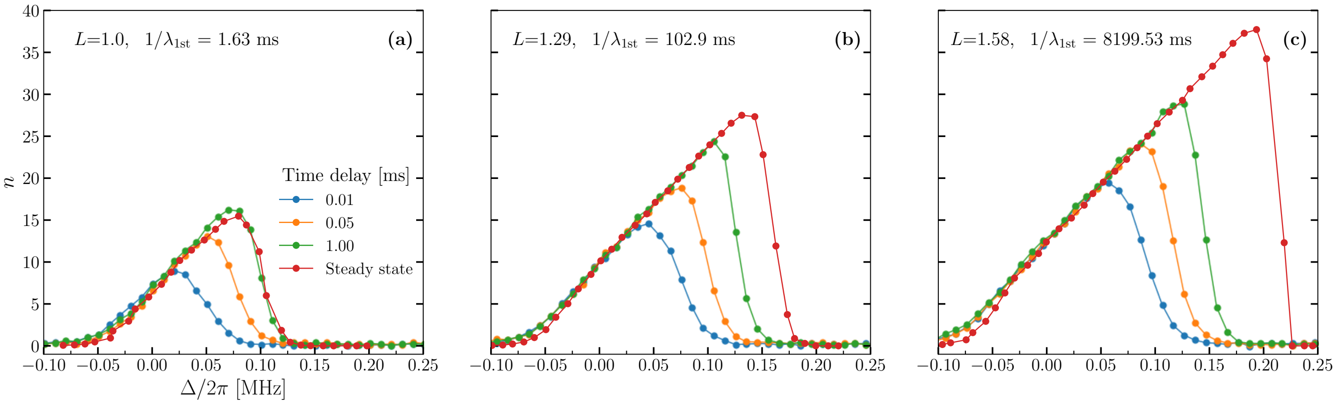

Supplementary Figure 1. **Photon number in the transient regime**. To show the importance of waiting an appropriately long time in order to measure the steady state, we sweep down the detuning from $\Delta = 2$ MHZ (deep in the vacuum) for (a) $L = 1$, (b) $L = 1.29$, and (c) $L = 1.58$. For each point in the plot, we wait for the delay time indicated in the legend before decreasing the detuning (for further detail, see the explanation of the hysteresis curves in the main text). When such a delay time is significantly smaller than the inverse of the Liouvillian gap (i.e., the typical timescale to reach the steady state), the measured photon number is smaller $n_{\rm ss}$. This effect becomes more prominent when the Liouvillian gap becomes smaller, which happens for larger values of L.

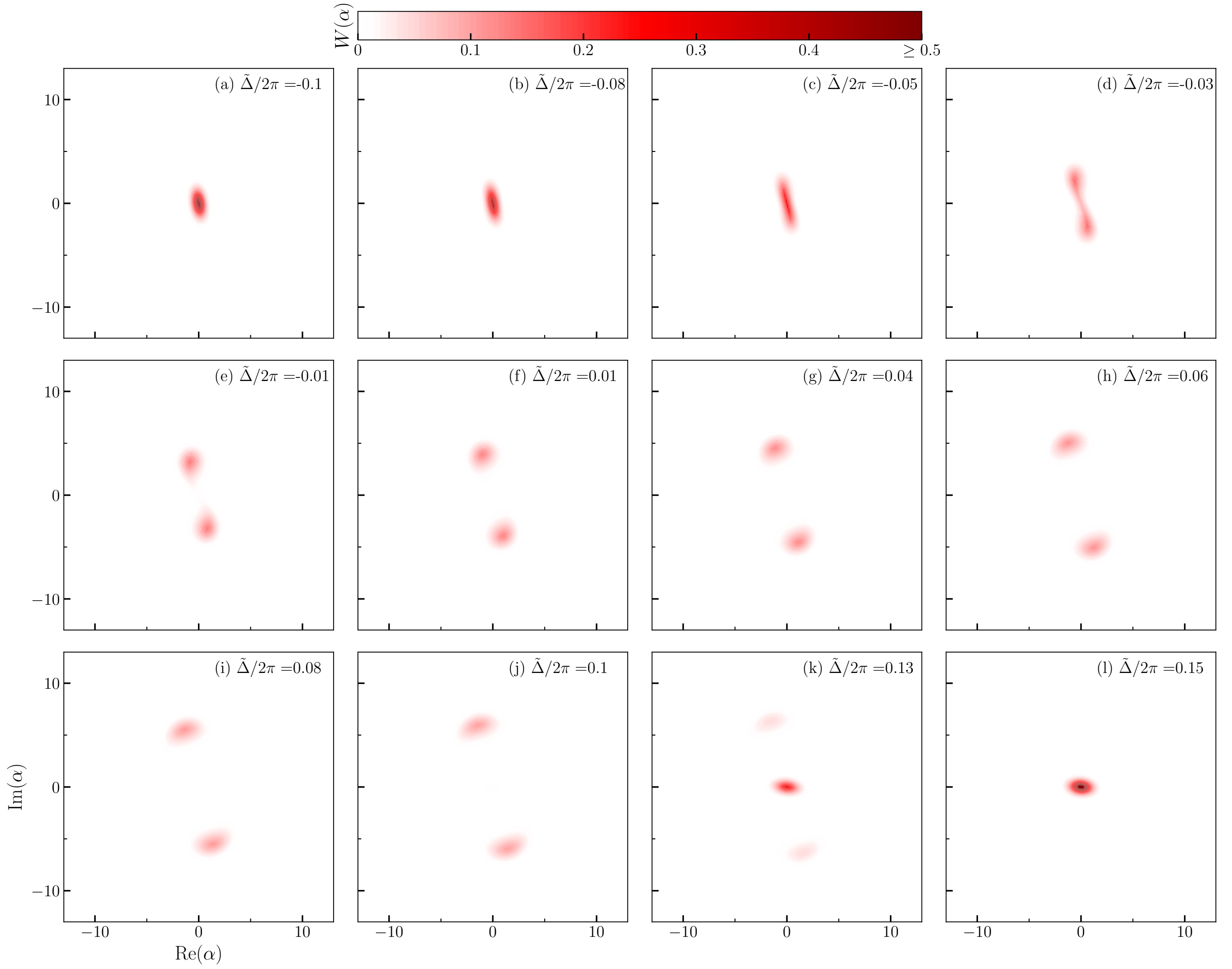

Supplementary Figure 2. **Theoretical Wigner functions**. We plot the theoretical Wigner function across the phase diagram for $L = 1.58$. The Wigner function was simulated based on the parameters fitted from the photon number and both $\lambda_{1\text{st}}$ and λ_{SSB}. The parameters used are the same as those in Fig. 2 of the main text.

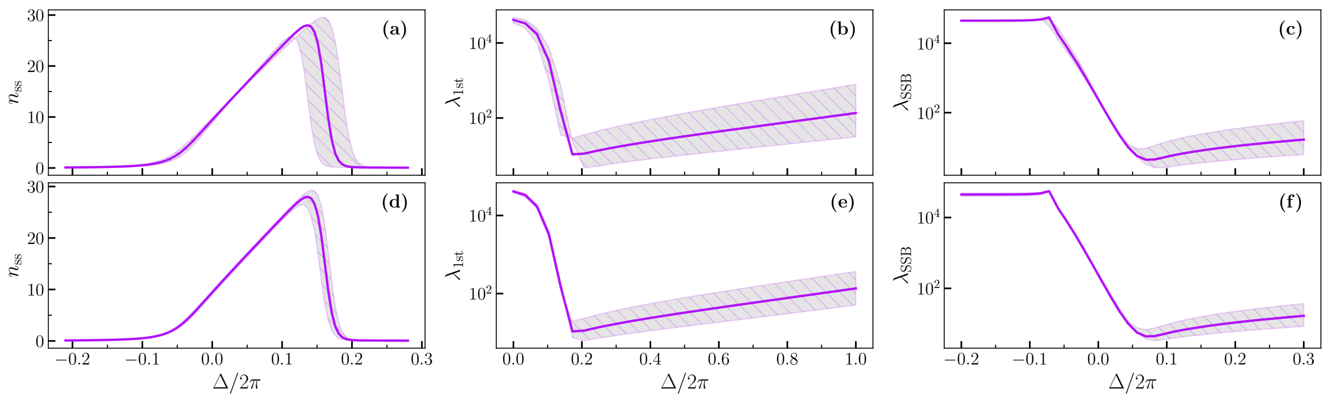

Supplementary Figure 3. **Numerical results upon a small change of the parameters.** In this figure we use Eq. (48) to derive (a,d) The photon number; (b,e) The Liouvillian eigenvalue λ_{1st}; and (c,f) The Liouvillian eigenvalue λ_{SSB}. The solid line indicates the results obtained for parameters used Figs. 2, 4, and 5 of the main text and $L = 1.29$. The shaded area encloses the numerical results: (a-c) for U and G are both increased and decreased by 10% relative to the values used to compute the solid line; (d-f) same for κ. Far from the transition, these variations do not significantly impact the data. However, they lead to noticeable differences in the critical region, particularly in the case of the Liouvillian eigenvalues.

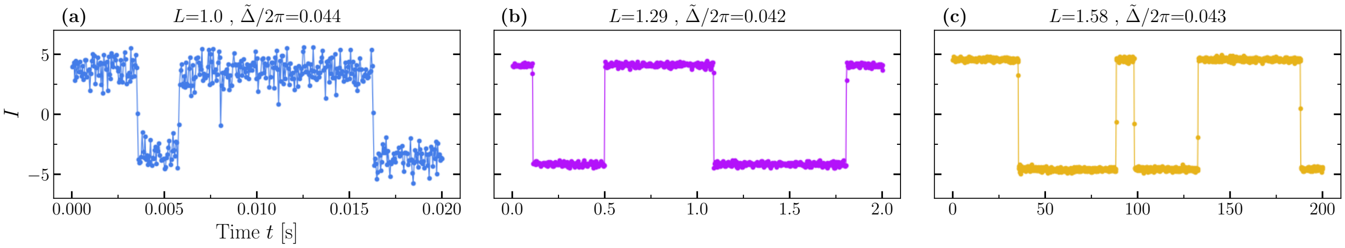

Supplementary Figure 4. **Sample of the measured heterodyned signal with different L.** As a function of time, we plot the real part of the cavity field quadrature I for approximately the same rescaled detuning. The colors indicate the different values of L as in Fig. 4 in the main text. Notice that the three panels present different x-scales. Random jumps between two opposite values of the quadrature occur as time passes and the rate of jumps is orders of magnitude slower than the photon-loss rate κ.

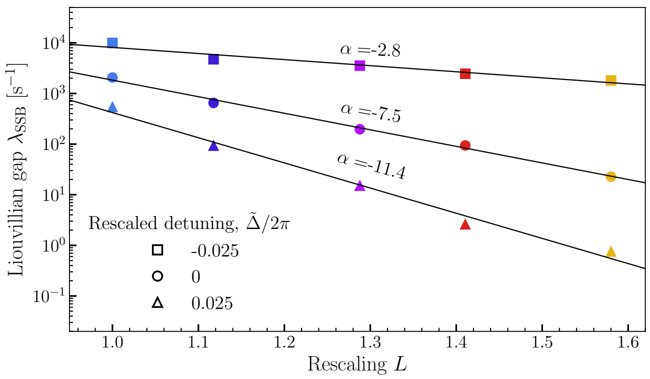

Supplementary Figure 5. **Scaling of the SSB Liouvillian gap at different detuning.** As a function of L, we plot the Liouvillian gap λ_{SSB} shown in Fig. 4(c) of the main text for different values of the rescaled detuning $\tilde{\Delta}$. The scaling rate strongly depends on the choice of $\tilde{\Delta}$, supporting the conclusions on the use of detuning as a resource for quantum computing discussed in [1–3].

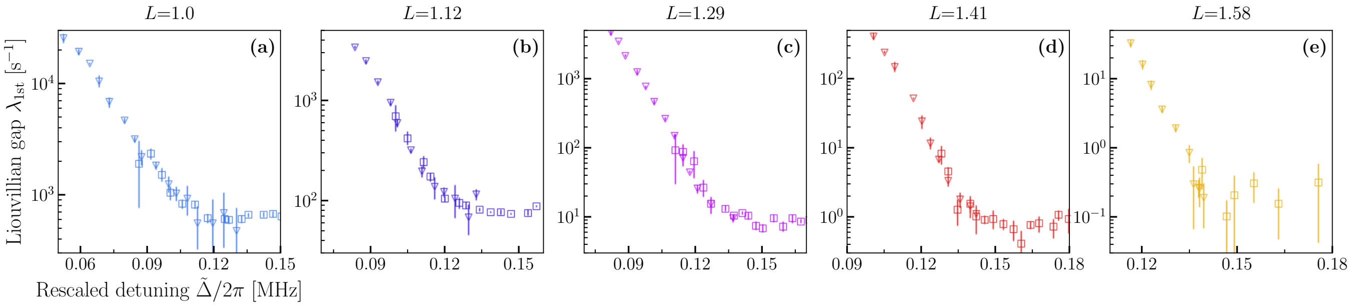

Supplementary Figure 6. **Zoom on the Liouvillian gap at the first-order DPT.** For the same L considered in the main text, and with the same color code, zoom on the minimum of Liouvillian gap shown in Fig. 5. Both methods of extrapolating the Liouvillian gap (starting from vacuum, triangle markers; starting from the bright phase, crosses) coincide at the minimum.

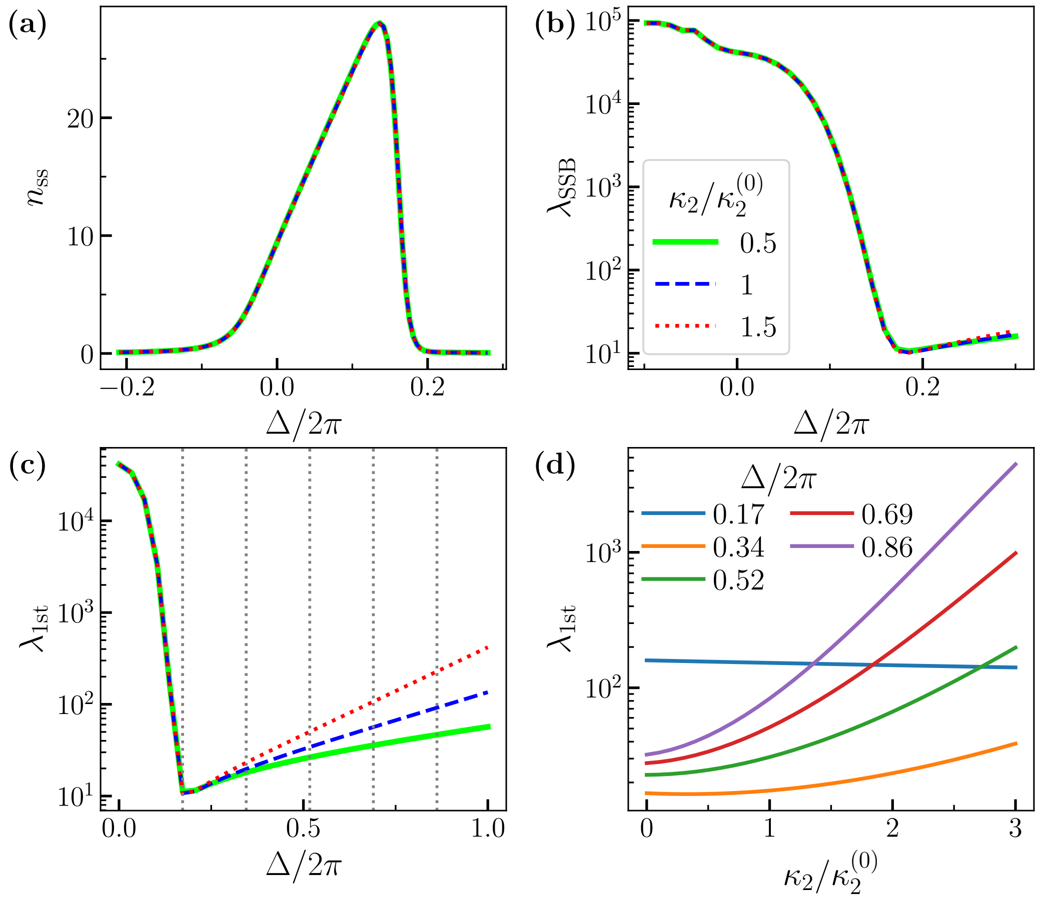

Supplementary Figure 7. **Effect of two-photon dissipation.** For $L = 1.29$, we numerically simulate the effect of changing κ_2 with respect to the original value $\kappa_2^{(0)} = 78$ Hz used in all other figures. While (a) the photon number and (b) λ_{SSB} are only marginally affected by a change in κ_2, (c) λ_{1st} is particularly sensitive to its value, especially at large detuning where the bright phase is metastable. (d) This dependence becomes more pronounced as the detuning is increased. The chosen detunings in (d) correspond to the vertical dashed lines in (c). We conclude that, in Kerr-dominated regimes where $\kappa_2 \ll U$, measuring the Liouvillian gap could be used as an efficient way to assess the value of κ_2.

Supplementary Note 2. SETUP AND DEVICE

A. Device design

Figure 8(a) shows an optical micrograph of the sample used in the experiment. The main component of the circuit is 6.18 mm long $\lambda/4$ resonator, made flux-tunable by terminating one end to ground via a DC superconducting quantum interference device (SQUID) [4]. The SQUID consists of two identical tunnel junctions designed to have an area of (0.75 x 0.75)µm^2. To drive the resonator parametrically, an L-shaped flux line is inductively coupled to the SQUID [see Fig. 8(d)]. As shown Fig. 8(b), the other end of the resonator is capacitively coupled to a feedline, which in our measurements is used solely to collect the emitted signal. After conducting the measurements, the feedline is repurposed for sending a single photon-drive to extract the device parameters, as discussed in Supplementary Sect. Supplementary Note 2 C. All waveguides are coplanar with a 16.47 µm wide centerline separated from the ground plane by a 10 µm gap, resulting in a characteristic impedance of approximately 50 Ω.

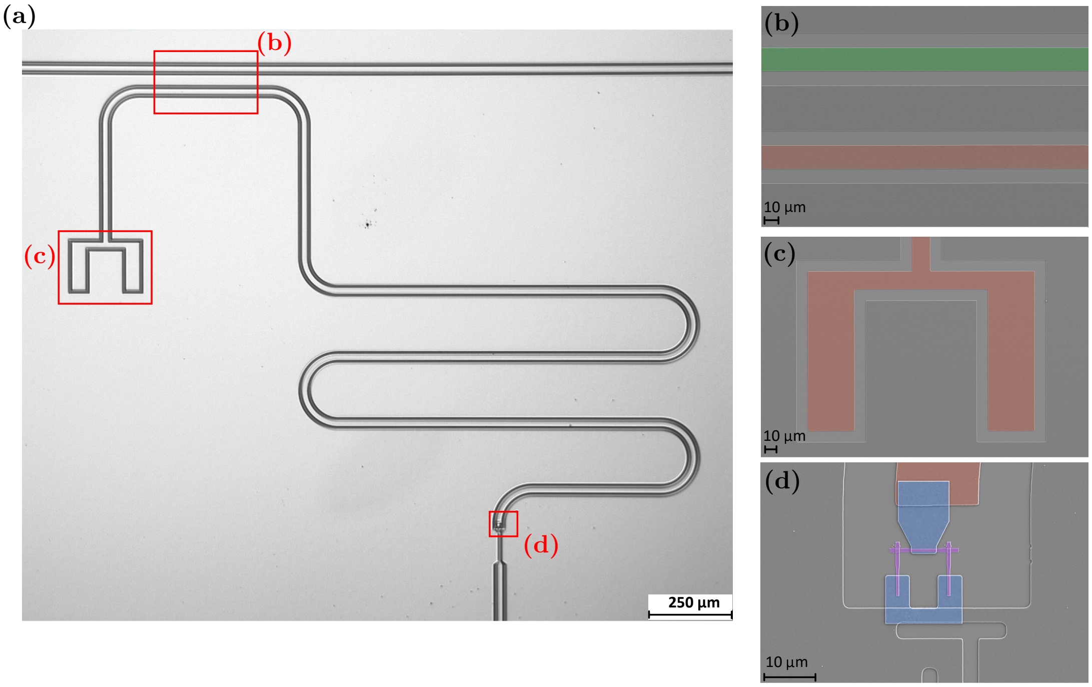

Supplementary Figure 8. **Optical and SEM images of the device** (a) Micrograph of the device: a $\lambda/4$ resonator terminated by a SQUID on one end and capacitively coupled to a feedline on the other. The three regions indicated by the red squares correspond to the locations where SEM images were taken. (b) Capacitive coupling between the feedline (green) and the resonator (red). The spacing between the centers of the two coplanar wave guides is 70 µm. (c) Open end of the resonator (red), designed for coupling to other elements in future experiments. (c) SQUID formed by two Josephson junctions (purple) galvanically connected to the resonator (red) by a patch (blue). The upper part of the fluxline is visible beneath the lower patch.

B. Device fabrication

The waveguides are made of a 150 nm thick aluminium layer deposited by e-beam evaporation (rate of 0.2 nm/s under a vacuum of $\sim 1.1 \times 10^{-6}$ Torr) onto a 525 µm thick silicon substrate. Prior to Al deposition, the substrate is thoroughly cleaned of organic residues using a piranha solution, and the native oxide is fully etched using 1% hydrofluoric acid (HF). During the first patterning step, alignment marks are defined by a photolithography and lift-off process. These marks are deposited by e-beam evaporation of a 5 nm thick layer of Ti followed by a 55 nm thick layer of Pt. Next, the waveguides are defined via photolithography and wet etching (2min30s in TechniEtch Alu80 at 27 °C). The Al/AlOx/Al Josephson junctions forming the SQUID are fabricated through the following steps: first, a bilayer resist (500 nm of MMA EL9 and 450 nm of PMMA 495k A8 developed in a 3:1 MiBK solution for 2min) is exposed using e-beam lithography, then Al is e-beam evaporated using the double-angle technique under

ultra-high vacuum (UHV) inside a Plassys MEB550SL3, and finally, a lift-off procedure is performed. The top and bottom Al layers of the junctions are deposited at a rate of 0.5 nm/s to achieve respective thicknesses of 50 nm and 120 nm. The junction barrier is grown during a static oxidation step carried out under a pressure of 0.15 Torr in pure O_2 atmosphere for 10 minutes. To connect the SQUID to the resonator and to the ground plane, a 200 nm thick Al patch is deposited by e-beam evaporation (deposition rate of 0.5 nm/s) and patterned by e-beam lithography and lift-off [see Fig. 8(d)]. To ensure a good electrical contact between the patch and the resonator/junctions, in situ Ar ion plasma milling is used to remove the native Al oxide. Finally, the substrate is diced into chips of size 4 x 7 mm using a nicked bonded diamond blade. The chip is then bonded with Al wire to a custom printed circuit board, which is screwed to a copper mount. To prevent slotline modes, bridge bounds connecting the ground plane across the chip are also added.

C. Experimental setup

The packaged sample is mounted in a high-purity copper enclosure which is thermally anchored at the mixing chamber stage of a BlueFors dilution refrigerator with a base temperature of 10 mK. To generate a DC flux bias on the sample, a coil made of NbTi wire is screwed underneath the sample holder. Two high permeability metal cans provide shielding against external magnetic fields. A schematic of the cryogenic and room temperature measurement setup is shown in Fig. 9. An OPX+ and Octave modules are used to generate the single-photon (at $\omega \sim \omega_r$) and the two-photons drives (at $\omega_p \sim 2\omega_r$) respectively directed to the feedline and the pump line. These signals are produced by modulating the octave's local oscillators with the I/Q signals originating from the OPX+. Throughout the experiment, the local oscillator used for the single-photon drive remains off to prevent any leakage field in the feedline. It is turned on only before/after the measurements are completed to extract the device parameters. After exiting the Octave, the two drives are split using a 2-way power divider ZSPD-20180-2S. Half of the signal enters the fridge, while the other half is directed to a spectrum analyzer (Signal Hound USB-SA124B) to monitor the drive amplitude and compensate for any drift. The two input lines have 20 dB, 10 dB, and 10 dB attenuators respectively positioned at the 4 K, 800 mK, and 100 mK stages. At the base plate, the input of the feedline has a 40 dB attenuator to limit the Kerr shift, whereas the pump line has a 10 dB attenuator. Multiple filters are incorporated along the pump line to eliminate higher or lower harmonics of the driving field. The output signal, collected via the feedline, passes through two circulators (LNF 4-8 GHz Dual Junction Circulator) and travels in a NbTi low-loss superconducting line before being amplified by a 4-8 GHZ LNF High-Electron-Mobility Transistor (HEMT) amplifier at the 4K plate. The output signal is further amplified at room temperature using a low noise amplifier (Agile AMT-A0284) before being demodulated in the Octave and digitized in the OPX+. Additionally, two filters are also placed along the output path to eliminate any signal coming from the flux line at ω_p. The switches positioned at the base plate are used to connect to different devices inside the fridge. Additionally, the switch on the output line is also connected to a 50 Ω cryogenic termination which is used to perform a Planck spectroscopy experiment (see Supplementary Sec. Supplementary Note 2 E). To set a static flux bias, a DC source (Yokogawa GS200) is connected to the coil attached under the sample.

D. Characterization of the device parameters

The first step in the characterization of the device is measuring the scattering coefficient S_{21} at low power while varying the magnetic flux bias. The scattering response is measured using a Vector Network Analyzer (Rohde & Schwarz, ZNA26 series) connected between the feedline and output line. The magnetic flux is varied by adjusting the current sent from the Yokogawa GS200 source to the NbTi coil underneath the sample. For each magnetic flux value, the resonance frequency ω_r is determined by fitting the scattering response using Eq. (46) (see Supplementary Sec. Supplementary Note 4 B for the derivation of S_{21}). The result of this measurement is shown in Fig. 10(a). Using Eq. (25), the flux response of the resonance frequency is fitted to determine the ratio of the SQUID to the cavity inductance (participation ratio) $\gamma \approx 3.1 \times 10^{-2}$ and bare cavity resonance frequency (without SQUID) $\omega_{\lambda/4} \approx$ 4.5068 GHz.

All the measurements discussed in the main text were performed at a magnetic flux bias of $F \approx \pi/6$, corresponding to the black star in Fig. 10(a). Figure 10(b) shows the real (blue markers) and imaginary (red markers) part of the corrected scattering response $\tilde{S}_{21} = S_{21}/ae^{j\alpha}e^{-j\omega\tau}$ at this operating point for an input power of -163 dbm. The resonance frequency, as well as the external and internal coupling parameters, are determined by fitting the data with Eq. (25). Repeating the measurement 12 times, we obtain the values: $\omega_r = 4.3497$ GHz, $\kappa_{ext} = 60$ kHz and, $\kappa_{int} = 17$ kHz. The value of ω_r presented here is obtained from measurements taken within a few minutes of interval. However, the resonance frequency is characterized by small fluctuations over long period of time. These fluctuations pose no problem to the experiment, as the relevant quantity to characterize the resonator is not ω_r, but rather the

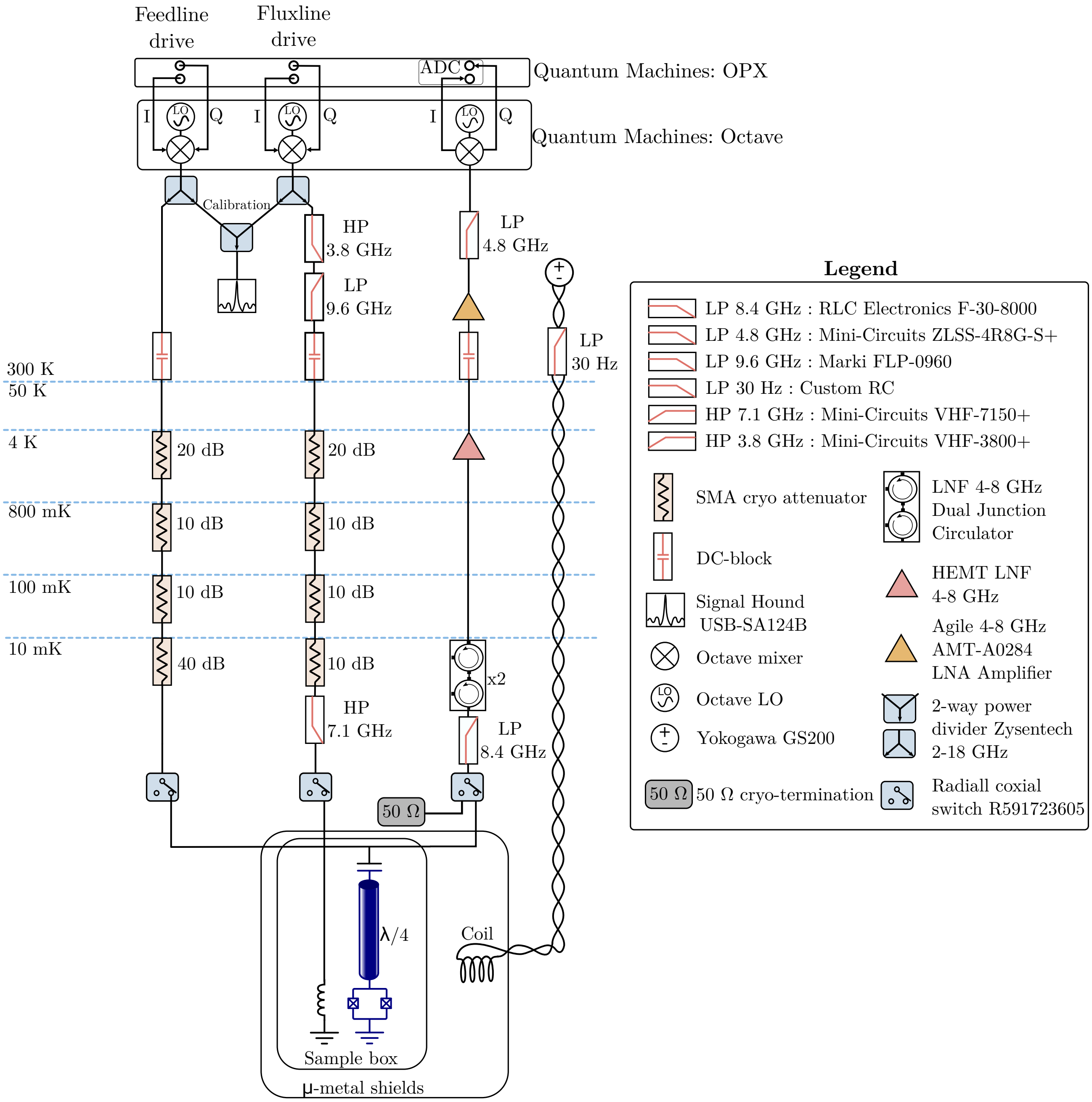

Supplementary Figure 9. **Schematic of the experimental setup.** A two photon-drive is generated by sending a signal to the flux line, which modulates the magnetic flux in the SQUID at frequency $\omega_p \sim 2\omega_r$. The driving field in the fluxline is generated by mixing (upconversion) the intermediate frequency signals I and Q from the OPX+ with one of the octave's local oscillator (set at 8.6 GHz). The parametric excitation of the cavity results in the emission of a signal at $\omega_p/2$, which is subsqeuntly amplified, and filtered to remove any component at ω_p. The output signal is mixed (downconversion) with another local oscillator of the octave (set at 4.3 GHz) to obtain the two quadratures I and Q at an intermediate frequency. These intermediate frequency signal are demodulated and integrated within the OPX+ over a time interval τ_{int}, resulting in a single pair of I and Q expressed in volts.

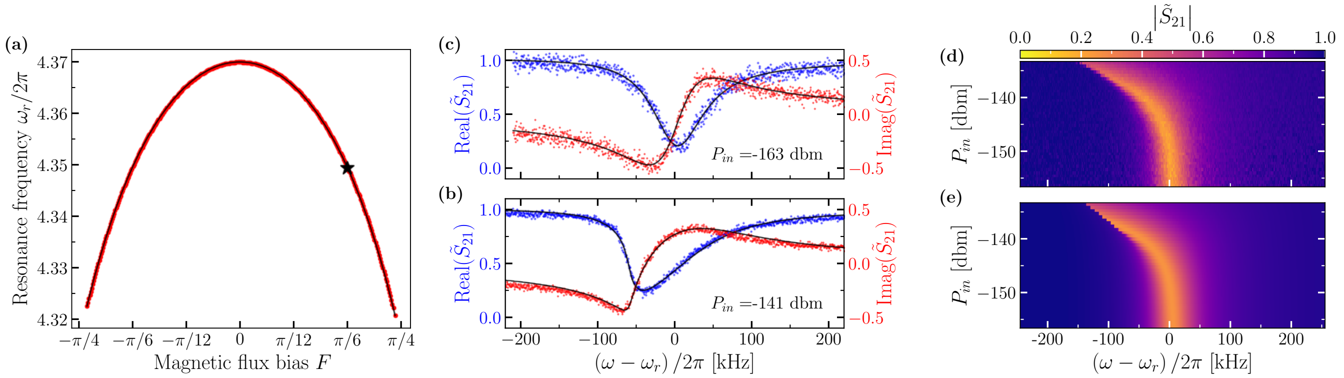

Supplementary Figure 10. **Measurement of the device parameters.** (a) Red markers show the measured resonance frequency ω_r for varying magnetic flux bias F. The solid black line indicates the fit to Eq. (25). The black star marks the operating point $F \approx \pi/6$ used throughout the experiment. (b)-(c) Real (blue) and imaginary (red) part of the measured rescaled scattering coefficient $\tilde{S}_{21} = S_{21}/ae^{j\alpha}e^{-j\omega\tau}$ at low (b) and high (c) input power. Notice that for high input power, the simple Lorentzian response observed at low power becomes distorted due to the nonlinearity of the resonator. The black lines indicate the fit to Eq. (46). (d) Absolute value of the measured rescaled scattering coefficient for increasing input power, and (d) the corresponding 2D fit to Eq. (46).

detuning between the pump and cavity. As briefly discussed in Sec. Supplementary Note 3, the detuning can be straightforwardly corrected before each experiment.

The Kerr nonlinearity U can be directly calculated from the measured participation ratio and bare resonance frequency using Eq. (35). Neglecting the SQUID capacitane ($C_J = 0$), a value of $U = 7\,\text{kHz}$ is obtained. The derivation of this equation and the validity of the approximation are detailed in Sec. Supplementary Note 4 A.

A second method to estimate the Kerr nonlinearity is fitting the scattering response S_{21} at higher power, where the nonlinearity influences the scattering coefficient (see Sec. Supplementary Note 4 B). The absolute value of the measured scattering coefficient as a function of the input power at the device P_{in} is shown in Fig. 10(d). Performing a 2D fit using Eq. (46), a value $U = 6\,\text{kHz}$ is obtained, thus confirming our initial estimate and the accuracy of our calibration. The fit is shown for the all the input powers in Fig. 10(e), and in Fig. 10(c) for a specific power of $P_{in} = -141$ dBm. The values experimentally extracted for the circuit parameters are summarized in Table 1.

Supplementary Table 1. **Experimental values of the circuit parameters**

$\omega_r/2\pi$ [GHz]	$\omega_{\lambda/4}/2\pi$ [GHz]	γ	$U/2\pi$ [kHz]	$\kappa_{ext}/2\pi$ [kHz]	$\kappa_{int}/2\pi$ [kHz]
4.3497	4.5068	3.13×10^{-2}	-7	60	17

E. Calibration of Input Attenuation, Amplifier Noise, and Gain

The two-photon pump populates the cavity. The signal emitted from the cavity, due to the emission into the waveguide at a rate κ_{ext}, passes through filters, amplifiers (cryo and room temperature) and cables before being collected. The power gain $\mathcal{G}$ of the output line takes into account all of these components, and relates the field measured at room temperature $\hat{c}(t)$ to the output field of the cavity at cryogenic temperature $\hat{b}^{(r)}_{out}(t)$ through the relation

$$\hat{c}(t) = \sqrt{\mathcal{G}}\,\hat{b}^{(r)}_{out}(t) + \sqrt{\mathcal{G}-1}\,\hat{h}^\dagger(t). \tag{1}$$

where the mode $\hat{h}(t)$ is a white noise (i.e., $\langle \hat{h}(t)\hat{h}^\dagger(t')\rangle = (n+1)\delta(t-t')$) that comes from a combination of cable losses, amplifier noise, and IQ mixer noise, and usually follows Gaussian statistics. For a given temporal filter $w(t)$ with normalization $\int dt\,|w(t)|^2 = 1$, one can consider the mode $\hat{c} = \int dt\, w(t)\hat{c}(t) = \int d\omega\,\tilde{w}(f)\hat{c}(f)$, where $\tilde{w}(f)$ and $\hat{c}(f)$ are the Fourier transform of $w(t)$ and $\hat{c}(t)$. The input-output relations for the filtered fields are

$$\hat{c} = \sqrt{\mathcal{G}}\,\hat{b}^{(r)}_{out} + \sqrt{\mathcal{G}-1}\,\hat{h}^\dagger, \tag{2}$$

where $\hat{b}^{(r)}_{out} = \int dt\, w(t)\hat{b}^{(r)}_{out}(t)$ and $\hat{h} = \int dt\, w(t)\hat{h}(t)$.

We perform a Planck spectroscopy experiment to calibrate $\mathcal{G}$ and the power n of the mode $\hat{h}$, i.e., $n = \langle \hat{h}^\dagger \hat{h} \rangle$ [5]. Using the switch positioned a the MXC stage of the cryostat, a $50\,\Omega$ cryogenic termination is connected to the output line (see Sec. Supplementary Note 2 C). This termination, thermalized at the MXC temperature T, acts as a black body emitter with average number of photons at frequency f given by $\bar{n}_T(f) = 1/\left\{\exp[hf/(k_B T)] - 1\right\}$. Therefore, the gain of the line can be calculated by comparing the theoretically known emitted power of thermal radiation from the attenuator in a bandwidth B, i.e., $P_T = \bar{n}_T(f)hfB$, to the measured power at room temperature P_m within the same bandwidth (i.e., we integrate with a filter $\tilde{w}(f) = 1_{f\in[f_0-B/2, f_0+B/2]}$).

To raise the temperature of the attenuator, the turbo pump of the cryostat is turned off, reducing the flow of the ^{3}He/^{4}He mixture, and consequently, the cooling power. Simultaneously, heat is applied through a heater at the MXC stage. The emitted power is given by

$$P_m = \frac{\langle \hat{I}_m^2 + \hat{Q}_m^2 \rangle}{Z_0} = B\mathcal{G}hf\left[\frac{1}{2}\coth\left(\frac{hf}{2k_B T}\right) + n\right], \quad (3)$$

where $k_B = 1.38\times10^{-23}$ J/K is the Boltzmann constant, $h = 6.63\times10^{-34}$ J s is the Planck constant. Fig. 11 shows the measured output power as a function of T. Here, $\hat{I}_m = \int_{f_0-B/2}^{f_0+B/2} df\ \hat{I}_m(f)$ and $\hat{Q}_m = \int_{f_0-B/2}^{f_0+B/2} df\ \hat{Q}_m(f)$, where $\hat{I}_m(f)$ and $\hat{Q}_m(f)$ are the Fourier transform of $\hat{I}_m(t)$ and $\hat{Q}_m(t)$, which are the quadratures of $\hat{c}(t)$ up to a dimensional normalization factor. The output power was measured at frequency $f = 4.3$ GHz (near the resonance frequency) in a bandwidth of $B = 5$ kHz. To ensure proper thermalization of the termination, we repeated the output power measurements at 10-minute intervals for each temperature setting until successive readings converged.

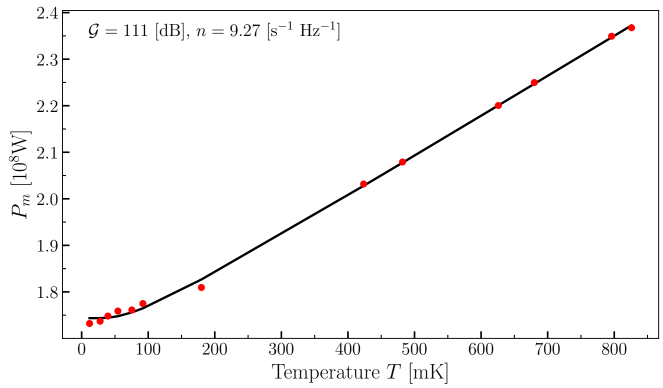

Supplementary Figure 11. **Gain calibration by Planck spectroscopy measurement**. (a) Blue markers indicate the measured output power at a frequency $f = 4.3$ GHz in a bandwidth $B = 5$ kHz as a function of the temperature. The solid black line is the fit with Eq. 3.

.

From the fit with Eq. 3, we obtain a power gain of $\mathcal{G} = 111$ dB and a mean photon noise of $n = 9.27\,\mathrm{s}^{-1}\,\mathrm{Hz}^{-1}$ corresponding to a noise temperature of 1.91 K.

Additionally, knowing the gain factor $\mathcal{G}$ of the output line allows for a straightforward characterization of the input attenuation of the feedline. The sample is used as a pass-through by applying a magnetic flux to shift its resonance frequency away from the measurement frequency. A known power P_d is then sent into the input line. This power is related to measured output signal power P_m by the following relation relation

$$P_m = \mathcal{G}\left[AP_d + Bhfn\right]. \quad (4)$$

We obtain an input attenuation $A = -85$ dB.

F. Moments reconstruction and squeezing parameter

For reconstructing the moments of $\hat{b}^{(r)}_{out}(t)$, we use the so-called reference-state method [6, 7]. This consists in reconstructing the moments of the noise mode $\hat{h}(t)$ using the vacuum as a reference state. Then, we use this characterization to retrieve the moments of the resonator output $\hat{b}^{(r)}_{out}(t)$. The method assumes a pre-knowledge of the measurement-line gain $\mathcal{G}$, which can be characterized with the method presented in the previous subsection.

We define the complex envelope operator

$$\hat{S} \equiv \frac{1}{\sqrt{Z_0 hf}} \frac{\hat{I}_m + i\hat{Q}_m}{\sqrt{\mathcal{G}}} = \hat{b}_{out}^{(r)} + \hat{h}^\dagger, \tag{5}$$

The real and imaginary parts of the complex envelope operator $\hat{S}$ represent the measured $\hat{I}_m$ and $\hat{Q}_m$ quadratures up to a normalization factor. The measured histograms and measurement traces are obtained from the quadratures of the complex envelope referred to the cavity

$$\hat{S}\sqrt{\frac{2}{\kappa_{ext}}} = \hat{I} + i\hat{Q} \tag{6}$$

As such, these quadratures are a convolution of the cavity field and amplifier noise. From the complex envelope operator, One can easily derive the relation [6, 7]

$$\langle (\hat{S}^\dagger)^n \hat{S}^m \rangle_{\rho_{\hat{b}_{out}^{(r)}}} = \sum_{i=0}^{n} \sum_{j=0}^{m} \binom{n}{i} \binom{m}{j} \langle (\hat{b}_{out}^{(r)\,\dagger})^i \hat{b}_{out}^{(r)\,j} \rangle \langle \hat{h}^{n-i} (\hat{h}^\dagger)^{m-j} \rangle. \tag{7}$$

Once the anti-normal ordered moments of the noise $\langle \hat{h}^n (\hat{h}^\dagger)^m \rangle$ is known, the set of linear equations in (7) can be solved for $\langle (\hat{b}_{out}^{(r)\,\dagger})^n \hat{b}_{out}^{(r)\,m} \rangle$. If $\hat{b}_{out}^{(r)}$ is in a vacuum state, Eq. (7) reduces to

$$\langle (\hat{S}^\dagger)^n \hat{S}^m \rangle_{|0\rangle\langle 0|} = \langle \hat{h}^n (\hat{h}^\dagger)^m \rangle, \tag{8}$$

since $\langle (\hat{b}_{out}^{(r)\,\dagger})^n \hat{b}_{out}^{(r)\,m} \rangle_{|0\rangle\langle 0|} = 0$ for $n, m \neq 0$. From Eq. (8) we can witness non-Gaussianity of the noise mode by looking at the cumulants [5]. We finally invert Eq. (7) for a generic input state. Notice that due to the increasing amount of terms involved for increasing n and m, higher moments reconstruction will have more statistical noise. However, the experimental samples are enough to reconstruct faithfully the moments up to $n + m = 2$. Let us write down the reconstruction formulas up to $n + m = 2$, as they are useful to characterize the squeezing:

$$\langle \hat{b}_{out}^{(r)} \rangle = \langle \hat{S} \rangle - \langle \hat{h}^\dagger \rangle \tag{9}$$

$$\langle \hat{b}_{out}^{(r)\,2} \rangle = \langle \hat{S}^2 \rangle - \langle (\hat{h}^\dagger)^2 \rangle - 2\langle \hat{b}_{out}^{(r)} \rangle \langle \hat{h}^\dagger \rangle \tag{10}$$

$$\langle \hat{b}_{out}^{(r)\,\dagger} \hat{b}_{out}^{(r)} \rangle = \langle \hat{S}^\dagger \hat{S} \rangle - \langle \hat{h}\hat{h}^\dagger \rangle - \langle \hat{b}_{out}^{(r)} \rangle \langle \hat{h} \rangle - \langle \hat{b}_{out}^{(r)\,\dagger} \rangle \langle \hat{h}^\dagger \rangle. \tag{11}$$

Again, non-Gaussianity of the mode $\hat{b}_{out}^{(r)}$ can be witnessed by computing higher moments, since Gaussian distributions are defined by the first-moment vector and the covariance matrix. So far we have discussed how to retrieve the moments of a filtered field $\hat{b}_{out}^{(r)}$. If one is interested in continuous monitoring of the moments of $\hat{b}_{out}^{(r)}(t)$, one can use a filter picked in t, i.e., consider $\frac{1}{\sqrt{\Delta T}} \int_{t-\Delta T/2}^{t+\Delta T/2} d\tau\ \hat{b}_{out}^{(r)}(\tau) \simeq \hat{b}_{out}^{(r)}(t)\sqrt{\Delta T}$.

With the output line calibrated, the intracavity field $\hat{a}(t)$ can be inferred with the input-output relations $\hat{b}_{out}^{(r)}(t) = \sqrt{\frac{\kappa_{ext}}{2}}\ \hat{a}(t) + \hat{b}_{in}^{(r)}(t)$ (Eq. 38 in Supplementary Sec. Supplementary Note 4 B). Consequently, substituting this relation in Eq. 5 in the absence of any input field in the feedline (i.e., setting $\hat{b}_{in}^{(r)}(t)$ to the vacuum), one can derive all the ordered moments of the integrated cavity mode $\hat{a} = \int dt\ w(t)\hat{a}(t)$, by inverting

$$\langle \hat{b}_{out}^{(r)\,\dagger k} \hat{b}_{out}^{(r)\,l} \rangle = \left(\frac{\kappa_{ext}}{2}\right)^{\frac{k+l}{2}} \langle \hat{a}^{\dagger k} \hat{a}^l \rangle. \tag{12}$$

Let us define the quadrature of $\hat{a}$ as $\hat{x}_\phi = \frac{1}{\sqrt{2}}(\hat{a}e^{-i\phi} + \hat{a}^\dagger e^{i\phi})$. One can use Eqs. (9)-(11) to reconstruct the variance $\langle \Delta x_\phi^2 \rangle = \langle \hat{x}_\phi^2 \rangle - \langle \hat{x}_\phi \rangle^2$. Notice that the quadrature variance of the vacuum state is set to 1/2.

For completeness, we show in Fig. 12 the anti-squeezed quadrature for $L = 1$. In Fig. 12 (a), we show the product of the squeezed (labeled Δx^2) and anti-squeezed (labeled Δp^2) quadrature variances. The product follows a clear trend. For large negative detuning, the state is in the vacuum and the Heisenberg inequality is saturated ($\Delta x^2 \Delta p^2 = 1/4$). Near the second-order critical point, the state is mixed, resulting in $\Delta x^2 \Delta p^2 \geq 1/4$. This is reflected in the large anti-squeezed variance. In Fig. 12 (b), we show the variance of the squeezed (black line) and anti-squeezed (red line) quadrature. The inset presents a zoom near the critical point, with the horizontal black line indicating 1/2. The detuning corresponding to the maximum $\partial^2 n_{\rm ss}$ is indicated by the dashed grey line in every panel.

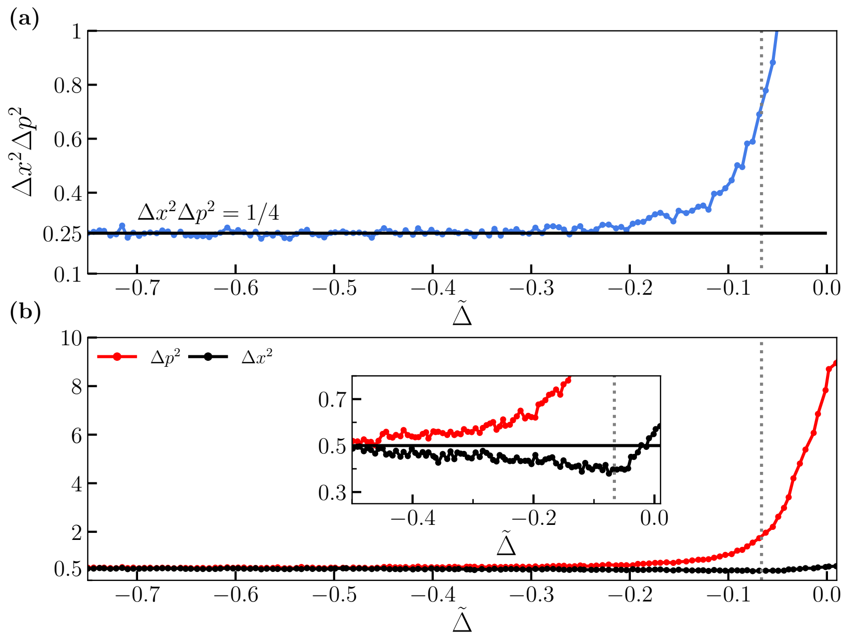

Supplementary Figure 12. **Squeezed and anti-squeezed variances**. (a) Product of the squeezed (Δx^2) and anti- squeezed quadrature variances (Δp^2) for $L = 1$ as a function of rescaled detuning $\tilde{\Delta}$. (b) The squeezed (black line) and anti-squeezed (red line) quadrature variances as a function of the rescaled detuning. The inset shows a zoom near the second-order critical point, with the horizontal black line indicating 1/2. The detuning corresponding to the maximum $\partial^2 n_{\rm ss}$ is indicated by the dashed grey line in every panel.

.

Supplementary Note 3. MEASUREMENT PROTOCOLS

Calibration of the cavity frequency: Prior to each measurement, the amplitude of the flux line signal is measured using the Signal Hound spectrum analyzer, and any amplitude drift is corrected. Furthermore, the resonance frequency is measured for every value of detuning during the experiment. This is necessary because the cavity resonance frequency slightly drifts over time, as a consequence of environmental noise (magnetic noise, vibrations in the lab, etc.). Figure 13 shows multiple measurements of the resonance frequency taken at intervals of 2 minutes over a period of $\sim$ 60 hours. The standard deviation over the full time interval is $\sim$ 4 kHz. This drift corresponds to a noticeable change in detuning and, if not accounted for, could lead to inconsistent results. Fortunately, since the relevant quantity in the experiment is the detuning, and not the cavity frequency alone, a drift in ω_r can be compensated by adjusting the pump frequency. To achieve this, we first evaluate the resonance frequency by measuring the scattering response S_{21} using a weak probe signal sent into the feedline, and fitting the result with Eq. (46). Then, the pump frequency is adjusted to obtain an accurate value of detuning. It is also important to note that the drift has negligible effect on the value of the Kerr nonlinearity [see Eq. (35)].

Acquisition of a measurement trace. After the calibration of the pump amplitude and detuning, each measurement involves three main steps: (I) initializing the system in the desired state, (II) activating the two-photon pump G at frequency ω_p, and (III) acquiring the measurement trace through heterodyne measurement. A single measurement trace is constructed from N quadrature measurements acquired sequentially, with a certain time delay τ_{delay} between each acquisition, while the two-photon pump is still on. Each quadrature measurement corresponds to the demodulated signal quadratures ($I_m(t)$,$Q_m(t)$) at frequency $\omega = \omega_p/2$, integrated over a time interval τ_{int} at the ADC of the OPX+. The quadratures ($I(t)$,$Q(t)$) of the intracavity field are then obtained by removing the effect of the amplification chain and its associated noise from the measured data (see Sect. Supplementary Note 2 E for more details). The details of each measurement, including the specific parameters used, are discussed below.

Dynamics at the first order DPT, i.e., measurement of λ_{1st}: To characterize the metastability of the vacuum, the system is initialized in the vacuum state, i.e., we set $G = 0$ which corresponds to no drive applied to the system. Then, the two-photon drive G is switched on at frequency ω_p. The corresponding the detuning is $\Delta = \omega_r - \omega_p/2$ (the latter having been adjusted for any frequncy drift). A measurement trace is then acquired following the procedure

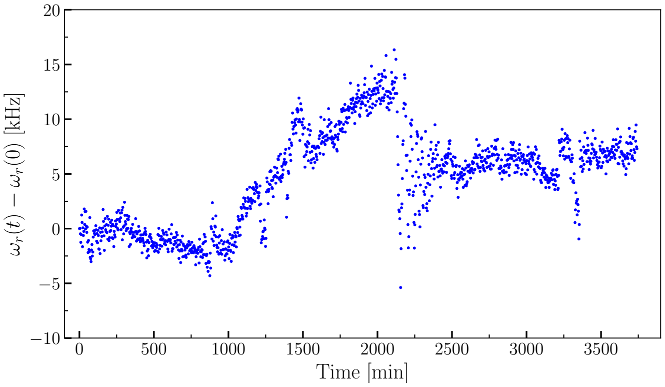

Supplementary Figure 13. **Resonance frequency drift.** Each marker, acquired every 2 minutes over $\sim$ 60 hours, corresponds to the resonance frequency extracted from the scattering response S_{21}.

described above. After measuring the trace, the two-photon drive is turned off and a waiting time τ_{wait} is required for the system to return to the vacuum state before the next measurement can be performed. For a given pump frequency ω_p (i.e., for a given detuning), the same measurement protocol is repeated M times giving M different measurement trace. The average over these traces is then fitted using

$$n(t) \simeq n_{\rm ss} + \delta n\, e^{-\lambda_{\rm 1st} t}, \tag{13}$$

to extract λ_{1st}. A schematic illustrating the measurement of the metastable vacuum is shown Fig. 14(a)-(b).

A similar protocol is followed to characterize the metastability of the bright phase, with the only difference being the initialization process. To initialize the system in the bright phase and observe the decay towards the vacuum, we apply the following protocol [see Fig. 14(d)] :

- The system is initialized in the vacuum state by choosing a pump frequency ω_p, such that the detuning is large and negative $\Delta \approx -0.3\,$MHz.
- Then, a strong two-photon drive of $G = 135$ kHz is turned on. For comparison, the maximal drive considered in the figures of the main text is $G = 103.5$ kHz.
- With the drive on, the detuning is continuously swept at a rate of 1 MHz/ms until the desired value of detuning is reached. Under these conditions, the system is in the bright state with a large number of photons.
- At this point, the two-photon drive is abruptly reduced to the desired value. The system then rapidly evolves to the desired bright metastable state.

This procedure ensures the system is in the bright phase at a given detuning and pump amplitude. The pulse sequence for this measurement is illustrated in Fig. 14(c) and the evolution of the system's state is also schematically represented in Fig. 14(d).

Since $\lambda_{\rm 1st}$ varies over several orders of magnitude depending on Δ and G, the measurement duration for each trace - determined τ_{int} and N - is calibrated based on an estimate of $\lambda_{\rm 1st}$ given by an initial sampling of 100 measurement traces. For $\lambda_{\rm 1st} < 100\,{\rm s}^{-1}$, the integration time is set to $\tau_{int} = 10\,\mu$s, while for $\lambda_{1st} > 100\,{\rm s}^{-1}$, $\tau_{int} = 50\,\mu$s. All measurements are done with a time delay $\tau_{delay} = 0$. The value of N ranged from $N = 1000$ to $N = 150000$, corresponding to a measurement time per trace varying from 0.01 s to 7.5 s. The choice of N is made such that the measurement time is at least twice $1/\lambda_{\rm 1st}$. However, at $L = 1.58$, for the values of detuning where the system is the slowest, the measurement time per trace is capped to 7.5 s in order to keep reasonable measurement times. The number of repetition is varied from $M = 500$ to $M = 125$ depending on the measurement time per trace. The experiment is repeated 4 times and error bars are obtained from the standard deviation over the experiments.

Dynamics at the second order, i.e., measurement of λ_{SSB}: Measuring λ_{SSB} involves recording a single very long measurement trace containing multiple jumps between the two coherent states $|\alpha\rangle$ and $|-\alpha\rangle$. Starting with the system in the vacuum state, the two-photon pump is switched on at a frequency ω_p and after a waiting time τ_{wait}, a measurement trace is acquired following the procedure described above. The auto-correlation function of the

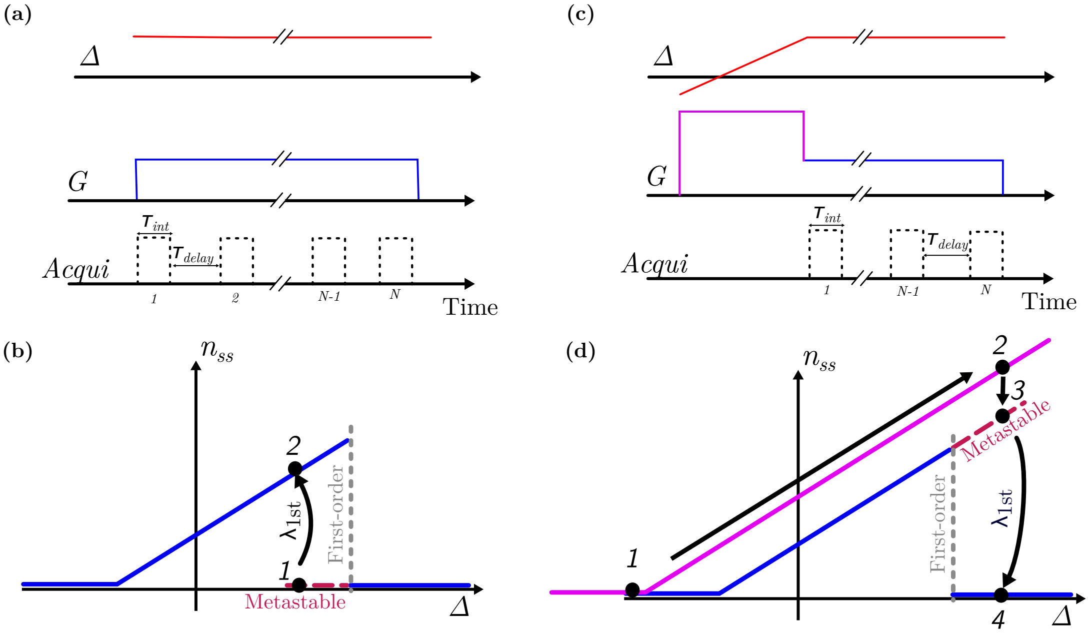

Supplementary Figure 14. **Pulse sequence for the measurement of λ_{1st}.** (a) The pulse sequence for measuring he vacuum metastability consists in activating the two-photon pump G (blue solid line) at a fixed detuning Δ (red solid line) and start acquiring the quadratures (dotted black line). The quadratures are acquired at time intervals τ_{delay} with each acquisition corresponding to the signal integrated over a time interval τ_{int}. A measurement trace consists of N acquisitions. (b) Schematic illustrating the measurement of vacuum metastability. The system is initialized in the vacuum state (black dot 1) by setting $G = 0$. After a certain time $\sim 1/\lambda_{1st}$, the system jumps to the steady-state bright phase (black dot 2). (c) The pulse sequence for measuring the bright phase metastability involves: (i) turning on a strong two-photon drive G (purple solid line) at large negative detuning Δ, (ii) increasing the detuning until the desired value is reached (red solid line), and (iii) abruptly lowering the value of G to reach the metastable bright phase (blue solid line). (d) Schematic illustrating the measurement of bright phase metastability. The bright phase initialization process begins by setting the system in the vacuum state at strong pump G (purple curve) and large netgative detuning (black dot 1). The detuning is then progressively increased until the desired value is reached (black dot 2), before abruptly lowering the pump to be in a metastable bright phase (black dot 3). After a certain time $\sim 1/\lambda_{1st}$, the system jumps to the steady-state vacuum (black dot 4).

measurement trace is then calculated and fitted using

$$C_{\mathrm{ss}}(t) = \sum_{n=1}^{N} \frac{C_n(\tau \gg 1/\kappa, t)}{N} \simeq \exp\{-\lambda_{\mathrm{SSB}} t\}. \tag{14}$$

to obtain λ_{SSB}. Since λ_{SSB} varies of several orders of magnitude depending on Δ and G, we first measure a few jump events to obtain an estimate of the duration - determined by τ_{int} and N and τ_{delay} - required to measure multiple jumps. For $\lambda_{SSB} < 100\,\mathrm{s}^{-1}$, the integration time is set to $\tau_{int} = 10\,\mu\mathrm{s}$, while for $\lambda_{SSB} > 100\,\mathrm{s}^{-1}$, $\tau_{int} = 50\,\mu\mathrm{s}$. The time delay τ_{delay} varied from zero to $1900\,\mu\mathrm{s}$ and the number of samples N ranged from 6.25×10^5 to 12.5×10^6, resulting in measurement times for a single measurement trace ranging from 12.5 s to 5 min. The waiting time τ_{wait} was kept constant for all measurements at 1 s. The experiment is repeated 4 times and error bars are obtained from the standard deviation over the experiments. For values of detuning and pump amplitude where $1/\lambda_{1st} > 1\,\mathrm{s}$, the initial samples in the trace, acquired before the system reached the steady state, were simply discarded.

Phase diagram, measurement of n_{ss} and Φ: The steady state properties can be directly inferred from the data used to calculate λ_{SSB}. The steady state photon number n_{ss} is obtained from averaging the quadrature squared over a single very long measurement trace $n_{ss} = \left\langle \hat{I}^2 + \hat{Q}^2 \right\rangle$. Note that near the second-order phase transition $1/\lambda_{SSB}$ becomes larger than τ_{int}. As a consequence, jumps occurring during the signal integration lead to a smaller value of n_{ss}. The phase of the system Φ corresponds to the angle of the complex number $I + iQ$ for one pair of quadratures. From the N pairs of quadratures in a single measurement trace, an histogram can be constructed representing the phase distribution during the measurement. Fig.1(e) is obtained by plotting such histograms as a function of the detuning Δ.

Squeezing measurement: The measurement of the squeezing follows a similar procedure to that of λ_{SSB}. The

key difference is that it requires faster measurements to avoid jumps occurring within the integration time of the quadrature. Additionally, a large number of samples are necessary to accurately reconstruct the state (see Sec. Supplementary Note 2 F for the state reconstruction process). The measurement traces used to calculate the squeezing are all acquired with the same parameters: an integration time $\tau_{int} = 2\,\mu s$, no delay $\tau_{delay} = 0$, a waiting time of $\tau_{wait} = 0.5\,s$ to reach the steady-state, and a total of $N = 10 \times 10^6$ samples.

Derivative calculation: The derivative calculation is performed using a finite-difference method over two points. Specifically, we first calculate the first derivative as follows:

$$\partial\tilde{n}_{\rm ss}(\tilde{\Delta}_i) = \frac{\tilde{n}_{\rm ss}(\tilde{\Delta}_{i+1}) - \tilde{n}_{\rm ss}(\tilde{\Delta}_i)}{\tilde{\Delta}_{i+1} - \tilde{\Delta}_i}, \tag{15}$$

where $\tilde{n}_{\rm ss}$ is the rescaled steady state photon number, $\tilde{\Delta}$ the rescaled detuning. The second derivative is then given by :

$$\partial^2\tilde{n}_{\rm ss}(\tilde{\Delta}_i) = \frac{\partial\tilde{n}_{\rm ss}(\tilde{\Delta}_{i+1}) - \partial\tilde{n}_{\rm ss}(\tilde{\Delta}_i)}{\tilde{\Delta}_{i+1} - \tilde{\Delta}_i}. \tag{16}$$

However, before performing these calculations, we first average over multiple points to reduce the noise in the derivative calculations. This procedure captures the overall trend of the derivatives smoothens the sharper features. In Fig.15 , we present the full analysis of the experimental data by plotting $\tilde{n}_{\rm ss}$ (first row) , $\partial_{\tilde{\Delta}}\,\tilde{n}_{\rm ss}$ (second row) and $\partial^2_{\tilde{\Delta}}\,\tilde{n}_{\rm ss}$ (third row) for different number of points averaged. The derivatives shown in the main text correspond to an averaged of 4 points.

Hysteresis measurement: To characterize the hysteretic behavior of the system, the detuning is ramped (by changing the pump frequency ω_p) while keeping the two-photon pump amplitude G constant. At each step of the detuning sweep, the quadratures are measured once, followed by a certain delay time τ_{delay} before moving to the next detuning value. Consequently, the detuning rate D is determined by the integration time τ_{int}, the delay time τ_{delay}, and the change in detuning in each pump frequency increment. The complete sweep is repeated M times, and the final result is obtained by averaging over all repetitions. The hysteresis maps shown in Figs.6(a,b) are the average of 4000 repetitions done with $\tau_{int} = 10\,\mu s$, $\tau_{delay} = 16\,ns$ and $D/2\pi = 1000\,MHz/s$. In Fig.6(c), a similar measurement is conducted to observe how the hysteresis area varies with the sweep rate. The sweep rate is changed from $D/2\pi = 1000\,MHz/s$ to $D = 35\,MHz/s$ by varying τ_{delay} from 16 ns to 0.28 ms. The integration time remains constant at $\tau_{int} = 10\,\mu s$ and the area is calculated from the average of $M = 4000$ repetitions.

Supplementary Note 4. MODELING OF THE SYSTEM

A. Hamiltonian parameters

The device is modeled by the following Hamiltonian

$$\hat{H}/\hbar = \omega_r\hat{a}^\dagger\hat{a} + \frac{U}{2}\hat{a}^\dagger\hat{a}^\dagger\hat{a}\hat{a} + \frac{G}{2}\left(\hat{a}^\dagger\hat{a}^\dagger e^{-i\omega_p t} + \hat{a}\hat{a}e^{i\omega_p t}\right), \tag{17}$$

with ω_r the resonance frequency, U the Kerr nonlinearity and G the two-photon drive amplitude. In the following, it will be explicitly demonstrated that this quantum mechanical model corresponds to a transmission line terminated by a SQUID as shown in Fig. 16(a). Through this calculation, equations for the system parameters ω_r and U will be derived. These equations provide valuable insights to design the device.

A transmission line of length d, with one end open and the other one grounded, forms a quarter wavelength resonator with eigenmode wavevectors $k_n = (\pi/d)(n + 1/2)$. The corresponding eigenmode frequencies are $\omega_n = (\pi/d\sqrt{lc})(n + 1/2)$, where l and c are respectively the inductance and capacitance per unit length [8]. The total inductance and capacitance of the cavity are simply expressed as $L_{cav} = ld$ and $C_{cav} = cd$. Adding a SQUID at the end of the transmission line ($x = d$) modifies the boundary condition, which results in an increase of the effective wavelength and, consequently, a deviation from the eigenmodes of a quarter wavelength resonator. To calculate this deviation, we follow the derivation of Refs. [8–10]. Throughout the following, emphasis will be placed on the fundamental mode, given its use in the experiment.

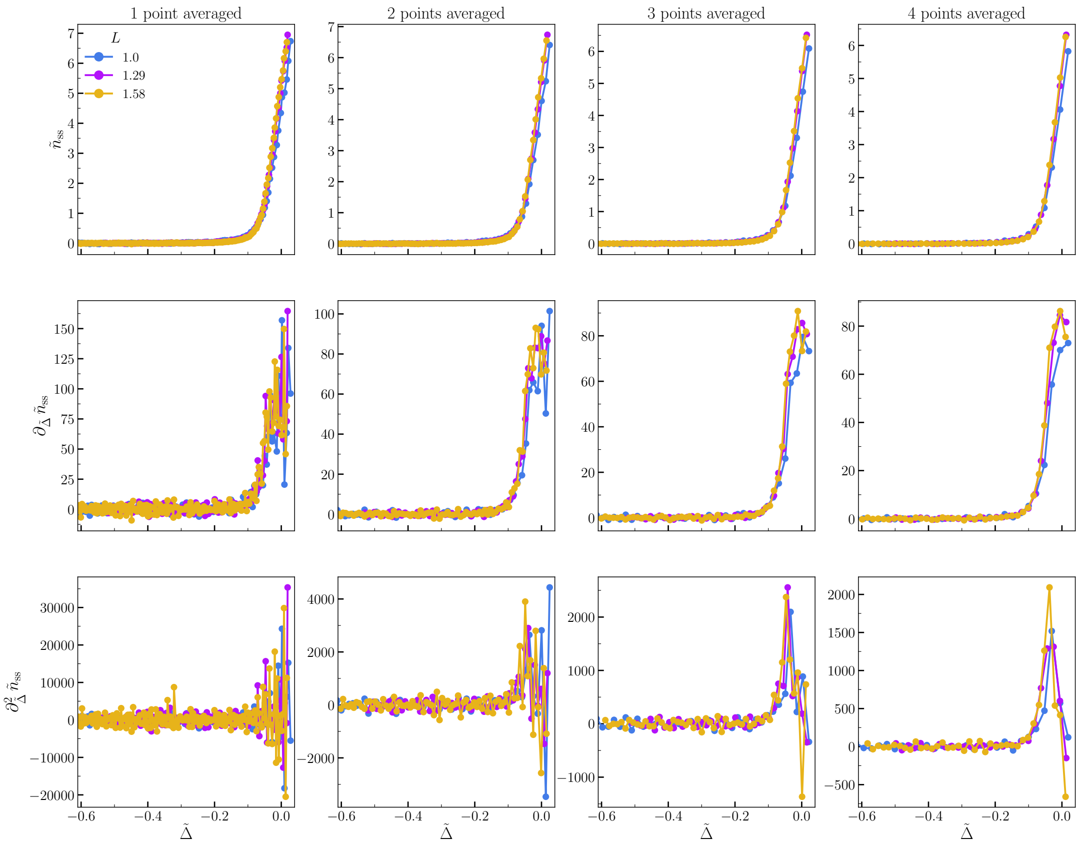

Supplementary Figure 15. **First and second derivative calculations for different number of points averaged**. $\tilde{n}_{\mathrm{ss}}$ (first row) , $\partial_{\tilde{\Delta}}\,\tilde{n}_{\mathrm{ss}}$ (second row) and $\partial^2_{\tilde{\Delta}}\,\tilde{n}_{\mathrm{ss}}$ (third row) for different number of points averaged. The calculation of $\partial_{\tilde{\Delta}}\,\tilde{n}_{\mathrm{ss}}$ and $\partial^2_{\tilde{\Delta}}\,\tilde{n}_{\mathrm{ss}}$ are performed according to Eqs.(15) and (16) respectively.

The total Lagrangian of the system [see Fig. 16(a)] is

$$\mathcal{L} = \underbrace{\int_0^d \left(\frac{c}{2}\dot{\phi}(x,t)^2 - \frac{1}{2l}\phi'(x,t)^2\right) dx}_{\text{Cavity}} + \underbrace{\frac{C_J}{2}\dot{\phi}(d,t)^2 + E_J \cos\left(\frac{\phi(d,t)}{\phi_0}\right)}_{\text{SQUID}}, \tag{18}$$

where C_J is the SQUID capacitance, E_J is the SQUID Josephson energy and $\phi_0 = \hbar/2e$ is the reduced flux quantum. Note that the two Josephson junctions forming the SQUID are considered to be identical with Josephson energy $E_{J,s}/2$. Under this condition, the SQUID can be modeled as a single junction with a tunable Josephson energy $E_J = E_{J,s}\,|\cos(F)|$, where F is given by the external magnetic flux enclosed by the SQUID loop. This also allows to represent the SQUID as a simple inductor of value $L_J = \phi_0^2/E_J$. The first term of Eq. (18), defines the equation of motion of cavity field

$$\ddot{\phi}(x,t) - v^2\phi''(x,t) = 0, \tag{19}$$

where $v = 1/\sqrt{lc}$ is the phase velocity. In addition to this wave equation, the cavity field is defined by two boundary conditions. Applying Kirchhoff current law at $x = d$ results in the first boundary condition

$$C_J\ddot{\phi}(d,t) + \frac{\phi_0}{L_j}\sin\left(\frac{\phi(d,t)}{\phi_0}\right) + \frac{\phi'(d,t)}{l} = 0. \tag{20}$$

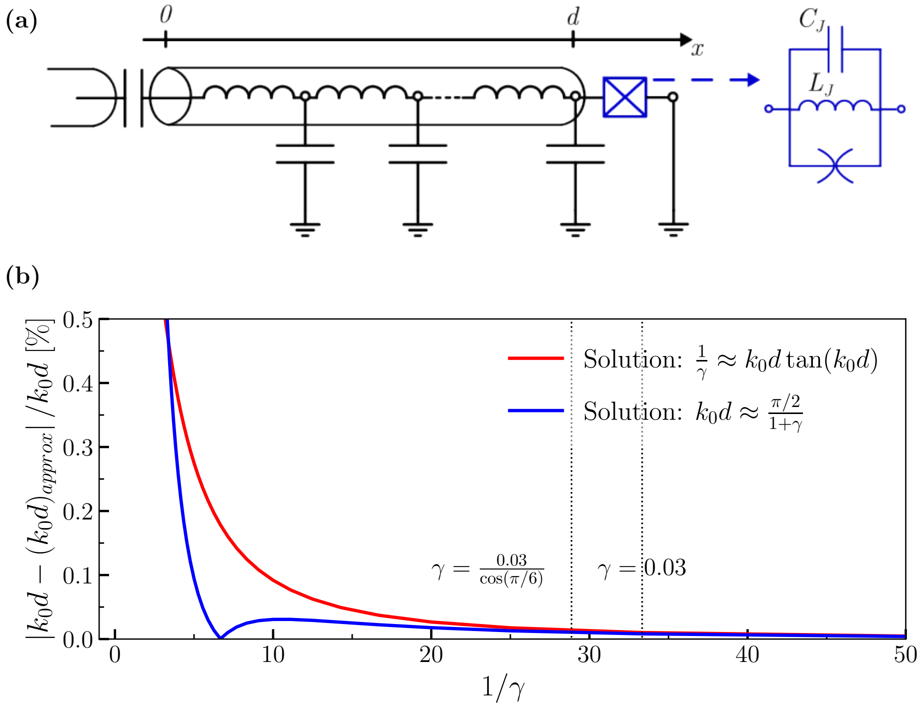

Supplementary Figure 16. **Distributed-element model of a $\lambda/4$ resonator terminated by a SQUID.** (a) The transmission line is modeled by a discrete chain of identical LC oscillators with inductance and capacitance per unit length of l and c respectively. The cavity has an open end at $x = 0$ and is grounded through a SQUID at $x = d$. (b) Relative error of the fundamental mode solution k_0 as a function of the inductance ratio. The red line is the solution of Eq. (22) neglecting the capacitance ratio, i.e. $C_J/C_{cav} = 0$, while the blue line is the first order approximation of Eq. (24). The relative error is calculated with respect to the solution obtained with Eq. (22) for $C_J/C_{cav} = 0.05$. The dotted line at $\gamma = 0.03$ corresponds to the participation ratio of our device without any applied external flux, while $\gamma = 0.03/\cos(\pi/6)$ is the participation ratio during the measurement.

At the open end of the cavity $x = 0$, the absence of current flow gives the second boundary condition

$$\frac{\phi(0,t)'}{l} = 0. \tag{21}$$

The latter condition fixes the form of the allowed eigenmodes, denoted by m, as

$$\phi_m(x,t) = \phi_m(t)\cos(k_m x). \tag{22}$$

Assuming a small amplitude for $\phi(d,t)$, Eq. (20) is linearized $[\sin(\phi(d,t)/\phi_0) \approx \phi(d,t)/\phi_0]$. Substituting Eq. (22) into this linearized boundary condition leads to

$$\frac{L_{cav}}{L_j} - \frac{C_J (k_m d)^2}{C_{cav}} = k_m d \tan(k_m d). \tag{23}$$

The solutions k_m to this transcendental equation determine the spatial degree of freedom of the eigenmodes of the system in the linear regime. The device used in the experiment was designed such that the capacitance and inductance ratio are respectively roughly $C_J/C_{cav} \approx 0.05$ and $\frac{L_J}{L_{cav}} \approx 0.03$. As will be shown below, the inductance participation ratio L_J/L_{cav}, denoted by γ, is a key design parameter. Neglecting SQUID capacitance ($C_J = 0$) and expanding Eq. (23) to the first order gives the following approximate solution for the fundamental mode

$$k_0 d \approx \frac{\pi/2}{1+\gamma}. \tag{24}$$

Expressed in terms of the resonance frequency $\omega_m = k_m v$, this can be rewritten

$$\omega_0 \approx \frac{\omega_{\lambda/4}}{1+\gamma}, \tag{25}$$

where $\omega_{\lambda/4}$ is the resonance frequency of the fundamental mode of the bare cavity (for $L_J = 0$) [11]. In Figure 16(b), the relative error of the fundamental mode solution k_0 is shown as a function of the inductance ratio. This comparison is made both when neglecting the SQUID capacitance (red curve) and when using the first-order solution of Eq. (25) (blue curve). The results clearly demonstrate, that within the parameter regime of the device, the capacitive term can be neglected, and that Eq. (25) is an excellent approximation.

Knowing the spatial solution of the eigenmodes, circuit quantization is performed by substituting $\phi_m(x,t)$ into the Lagrangian Eq. (18). Interactions between the eigenmodes are neglected.

$$\mathcal{L} = \int_0^d \left(\frac{c}{2} \dot{\phi}_m(t)^2 \cos^2(k_m x) - \frac{k_m^2}{2l} \phi_m(t)^2 \sin^2(k_m x) \right) dx + \frac{C_J}{2} \dot{\phi}_m(t)^2 \cos^2(k_m d) + E_J \cos\left(\frac{\phi_m(t)\cos(k_m d)}{\phi_0} \right). \quad (26)$$

Expanding the nonlinear potential to the second order: $E_J \cos\left(\frac{\phi_m(d,t)}{\phi_0}\right) \sim -\frac{E_J}{2\phi_0^2}\phi_m(d,t)^2 + \frac{E_J}{24\phi_0^2}\phi_m(d,t)^4$, gives

$$\begin{aligned}\mathcal{L} = \int_0^d \left(\frac{c}{2} \dot{\phi}_m(t)^2 \cos^2(k_m x) - \frac{k_m^2}{2l} \phi_m(t)^2 \sin^2(k_m x) \right) dx + \frac{C_J}{2} \dot{\phi}_m(t)^2 \cos^2(k_m d) \\ - \frac{E_J \phi_m(t)^2}{2\phi_0^2} \cos^2(k_m d) + \frac{E_J \phi_m(t)^4}{24\phi_0^4} \cos^4(k_m d).\end{aligned} \quad (27)$$

An effective LC oscillator can be defined from the linear part of Eq. (27). The oscillator as an effective capacitance C_m and inductance L_m defined as

$$C_m = c\int_0^d \cos^2(k_m x)dx + C_J \cos^2(k_m d) = \frac{C_{cav}}{2} M_m, \quad (28)$$

$$L_m^{-1} = \int_0^d \frac{k_m^2}{l} \sin^2(k_m x)dx + \frac{E_J}{\{\phi_0^2} \cos^2(k_m d) = \frac{(k_m d)^2}{2L_{cav}} M_m, \quad (29)$$

where,

$$M_m = \left[1 + \frac{\sin(2k_m d)}{2k_m d} + \frac{2C_J}{C_{cav}} \cos^2(k_m d) \right]. \quad (30)$$

Note that the equality of Eq. (29) is found by substituting $1/L_J$ by Eq. (23). Using the above definitions, the Lagrangian of Eq. (27) simplifies to

$$\mathcal{L} = \frac{C_m}{2} \dot{\phi}_m(t)^2 - \frac{1}{2L_m} \phi_m(t)^2 + \frac{E_j \phi_m(t)^4}{24\phi_0^4} \cos^4(k_m d). \quad (31)$$

A Legendre transformation of the Lagrangian (neglecting any mode interactions) results in the following Hamiltonian

$$\mathcal{H} = \frac{C_m}{2} q_m(t)^2 + \frac{1}{2L_m} \phi_m(t)^2 - \frac{E_j \phi_m(t)^4}{24\phi_0^4} \cos^4(k_m d), \quad (32)$$

where $q_m(t) = C_m \dot{\phi}_m(t)$ is the conjugate variable of $\phi_m(t)$. In the quantum regime $\phi_m(t)$ and $q_m(t)$ are operators satisfying the commutation relation $\left[\hat{\phi}_m, \hat{q_m}\right] = i\hbar$. These operators can be rewritten in terms of normal mode annihilation $\hat{a}$ and creation operators $\hat{a}^\dagger$

$$\begin{aligned}\hat{\phi}_m &= \phi_{zpf,m}(\hat{a}_m + \hat{a}_m^\dagger) \\ \hat{q}_m &= -iq_{zpf,m}(\hat{a}_m - \hat{a}_m^\dagger),\end{aligned} \quad (33)$$

where $q_{zpf,m} = \sqrt{\hbar\omega_m C_m/2}$ and $\phi_{zpf,m} = \sqrt{\hbar/2\omega_m C_m}$. Under the rotating wave approximation and considering only the fundamental mode $m = 0$, the Hamiltonian of the system can be expressed as

$$\hat{H}/\hbar = \omega_r \hat{a}^\dagger \hat{a} + \frac{U}{2} \hat{a}^\dagger \hat{a}^\dagger \hat{a}\hat{a}, \quad (34)$$

where there resonance frequency is $\omega_r = \omega_0 + U$ and Kerr nonlinearity is

$$U = -\frac{E_J}{2\hbar} \left(\frac{\phi_{zpf,0}}{\phi_0} \right)^4 \cos^4(k_0 d) = -\frac{\hbar\omega_0^2 L_{cav}}{2\gamma\phi_0^2} \left[\frac{\cos^2(k_0 d)}{(k_0 d)^2 M_0} \right]^2. \quad (35)$$

Note that, since $\omega_0 \gg U$, we can neglect the small photon number dependent frequency shifts due to the nonlinear term such that $\omega_r = \omega_0$.

B. Open-system parameters

To model the entire system, the Hamiltonian of the $\lambda/4$ Kerr resonator has to be modified to incorporate the interaction with the environment, i.e., the surrounding bosonic baths. The system is coupled to three distinct baths: the feedline, which is separated into right- and left-propagating modes, and the intrinsic bath. As we are interested in fitting the scattering coefficients (depending on the intracavity photon number), only the dominant effects of photon loss are considered. Following the approach of [12], the total Hamiltonian can be described as

$$\begin{aligned}\hat{H}/\hbar = &\underbrace{\omega_r \hat{a}^\dagger \hat{a} + \frac{U}{2}\hat{a}^\dagger\hat{a}^\dagger\hat{a}\hat{a}}_{\text{Cavity}} + \underbrace{\int d\omega\, \omega\, \hat{b}_\omega^{(int)\,\dagger}\hat{b}_\omega^{(int)} + i g_{int}\left(\hat{b}_\omega^{(int)\,\dagger}\hat{a} - \hat{b}_\omega^{(int)}\hat{a}^\dagger\right)}_{\text{Intrisic bath + coupling}} \\ &+ \underbrace{\int d\omega\, \omega\, \hat{b}_\omega^{(r)\,\dagger}\hat{b}_\omega^{(r)} + i g_r\left(\hat{b}_\omega^{(r)\,\dagger}\hat{a} - \hat{b}_\omega^{(r)}\hat{a}^\dagger\right)}_{\text{Right propagating modes + coupling}} + \underbrace{\int d\omega\, \omega\, \hat{b}_\omega^{(l)\,\dagger}\hat{b}_\omega^{(l)} + i g_l\left(\hat{b}_\omega^{(l)\,\dagger}\hat{a} - \hat{b}_\omega^{(l)}\hat{a}^\dagger\right)}_{\text{Left propagating modes + coupling}},\end{aligned} \tag{36}$$

where $\hat{b}_{int/l/r}$ are the harmonic oscillator modes associated with the internal, right- and left-propagating baths and $g_{int/r/l}$ represents the coupling strength between these modes and the resonator field $\hat{a}$. We assumed these couplings to be independent on the frequency of bath modes. Following standard input-output theory [12, 13], the time evolution of $\hat{a}$ is defined by the quantum Langevin equation

$$\dot{a}(t) = -i\omega_r \hat{a}(t) - iU\hat{a}^\dagger(t)\hat{a}^2(t) - \frac{\kappa_{int}+\kappa_l+\kappa_r}{2}\hat{a}(t) - \sqrt{\kappa_l}\hat{b}_{in}^{(l)}(t) - \sqrt{\kappa_r}\hat{b}_{in}^{(r)}(t) - \sqrt{\kappa_{int}}\hat{b}_{in}^{(int)}(t), \tag{37}$$

where $\kappa_{int/l/r} \to \sqrt{\frac{g_{int/l/r}}{2\pi}}$ and the input fields are defined as $\hat{b}_{in}^{(l/r)}(t) = \frac{1}{\sqrt{2\pi}}\int_{-\infty}^{\infty} d\omega e^{-i\omega t}\hat{b}_\omega^{(l/r)}(0)$. The input-output relations for the fields propagating in the feedline are

$$\begin{aligned}\hat{b}_{out}^{(l)}(t) &= \hat{b}_{in}^{(l)}(t) + \sqrt{\kappa_l}\hat{a}(t), \\ \hat{b}_{out}^{(r)}(t) &= \hat{b}_{in}^{(r)}(t) + \sqrt{\kappa_r}\hat{a}(t), \\ \hat{b}_{out}^{(int)}(t) &= \hat{b}_{in}^{(int)}(t) + \sqrt{\kappa_{int}}\hat{a}(t).\end{aligned} \tag{38}$$

Assuming an equal coupling between the intra-resonator mode and both the left- and right-propagating mode ($\kappa_l = \kappa_r$), we define the total external coupling κ_{ext} as $\kappa_{ext} = 2\kappa_r$. By Fourier transforming Eq. (37) and substituting for κ_{ext}, we obtain

$$i(\omega_r - \omega)\hat{a}(\omega) + iU\hat{a}^\dagger(\omega)\hat{a}^2(\omega) + \frac{\kappa_{int}+\kappa_{ext}}{2}\hat{a}(\omega) = -\sqrt{\frac{\kappa_{ext}}{2}}\left(\hat{b}_{in}^{(r)}(\omega) + \hat{b}_{in}^{(l)}(\omega)\right) - \sqrt{\kappa_{int}}\hat{b}_{in}^{(int)}(\omega). \tag{39}$$

To characterize the parameters of the system, only the right propagating input field is sent to the cavity, i.e., $\hat{b}_{in}^{(l)}(t) = 0$ and $\hat{b}_{in}^{(int)}(t) = 0$ are the vacuum mode. Furthermore, assuming that the intraresonator and input fields are coherent states, respectively defined as $\hat{a}\left|\alpha\right\rangle = \alpha\left|\alpha\right\rangle$ and $\hat{b}_{in}^{(r)}\left|\beta_{in}^{(r)}\right\rangle = \beta_{in}^{(r)}\left|\beta_{in}^{(r)}\right\rangle$, we can rewrite Eq. (39) as

$$i(\omega_r - \omega)\alpha + iU\left|\alpha\right|^2\alpha + \frac{\kappa_{int}+\kappa_{ext}}{2}\alpha = -\sqrt{\frac{\kappa_{ext}}{2}}\beta_{in}^{(r)}. \tag{40}$$

This approximation, known as the semiclassical approximation, is justified either in the limit in which $U\left|\alpha\right|^2 \ll \kappa_{ext}$, and it is known to be predictive for the Kerr resonator far from the critical points [14, 15].

Following Refs. [9, 16], we multiply Eq. (40) by its complex conjugate to derive the average photon number $\left|\alpha\right|^2$ in the resonator

$$\left(\delta^2 + \frac{1}{4}\right)n - 2\delta\xi n^2 + \xi^2 n^3 = \frac{1}{2}, \tag{41}$$

where the scale invariant quantities δ, ξ and n are defined as

$$\delta \equiv \frac{\omega - \omega_r}{\kappa_{in} + \kappa_{ext}}, \tag{42}$$

$$\xi \equiv \frac{\left|\beta_{in}^{(r)}\right|^2 \kappa_{ext} U}{\left(\kappa_{ext} + \kappa_{int}\right)^3}, \tag{43}$$

$$n \equiv \frac{|\alpha|^2}{\left|\beta_{in}^{(r)}\right|^2} \frac{\left(\kappa_{ext} + \kappa_{int}\right)^2}{\kappa_{ext}}. \tag{44}$$

After solving for n in the above equation, we can calculate the scattering parameter S_{21} from Eqs. (44) and (38) in terms of the scale invariant quantities (Eqs. (42), (43), (44))

$$S_{21} = \frac{\left\langle \hat{b}_{out}^{(r)} \right\rangle}{\left\langle \hat{b}_{int}^{(r)} \right\rangle} = 1 - \frac{\kappa_{ext}}{\kappa_{ext} + \kappa_{int}} \frac{1}{1 + 2j(\delta - \xi n)}. \tag{45}$$

Note that, to be consistent with other results in the literature [12, 16], we have written the scattering coefficient using the electrical engineering convention for the imaginary unit. In this convention, the imaginary unit is defined as $j = -\sqrt{-1}$, instead of the common physics convention in which the imaginary unit is $i = \sqrt{-1}$ [12, 17]. To perform a direct fit of the experimental data for S_{21}, it is necessary to introduce a correction factor that takes into account the net attenuation or gain of the line and the phase shift introduced by the finite speed of the field and the cable length. These corrections are done by multiplying Eq. (45) by $S_{env} = ae^{j\alpha}e^{-j\omega\tau}$, where a is an additional amplitude, α is a phase shift and τ is the electronic delay [18]. In addition, following the diameter correction method [19], we also introduce a factor of $e^{j\phi}/\cos\phi$ to compensate for any impedance mismatch. The corrected scattering coefficient is

$$S_{21} = ae^{j\alpha}e^{-j\omega\tau}\left(1 - \frac{\kappa_{ext}}{\kappa_{ext} + \gamma_{int}} \frac{e^{j\phi}}{\cos\phi} \frac{1}{1 + 2j(\delta - \xi n)}\right). \tag{46}$$

In the limit of low photon number ($n \to 0$), nonlinear effects are negligible and Eq. (46) can be fitted directly to extract κ_{ext} and κ_{int}. Note that the dephasing rates κ_ϕ and the losses through the flux line κ_F are all included in κ_{int} by this approximation. At higher input power, one must first solve for n using Eq. 44, and subsequently substitute into Eq. 46. Fitting at higher input power fit allows us to extract ξ from which we can either deduce U. However, this requires knowing the incoming photon flux

$$\left|\beta_{in}^{(r)}\right|^2 = \frac{10^{(P_d + A)/10}}{\hbar\omega} 10^{-3}, \tag{47}$$

with P_d the power in dbm at room temperature and A is the attenuation of the input line.

Supplementary Note 5. PARAMETER ESTIMATION

All through the work, we use the model

$$\frac{\partial \rho}{\partial t} = -\mathcal{L}\rho = -\frac{i}{\hbar}[\hat{H}, \rho] + \kappa(n_{\rm th} + 1)\mathcal{D}[\hat{a}]\rho + \kappa n_{\rm th}\mathcal{D}[\hat{a}^\dagger]\rho + \kappa_\phi \mathcal{D}[\hat{a}^\dagger \hat{a}]\rho + \kappa_2 \mathcal{D}[\hat{a}^2]\rho, \tag{48}$$

to perform our numerical simulation. To study the photon number, we compute the steady state by numerically solving the system $\mathcal{L}\rho_{\rm ss} = 0$. For the computation of the Liouvillian gaps, instead, we block-diagonalize the Liouvillian [20] and perform an Arnoldi-iteration algorithm using shifted-inverted strategy to find the minimal eigenvalue. For each simulation, convergence in the cutoff is verified by confirming that, increasing the size of the Hilbert space, data are within 1% difference.

As detailed in the main text, the parameters κ_ϕ, κ_2, G, and $n_{\rm th}$ cannot be directly argued from straightforward measures, such as those described in Supplementary Note 4 B. We thus resort to an optimization strategy, aimed at reproducing the experimental curves for the photon number $n_{\rm ss}$, as well as the Liouvillian gaps $\lambda_{\rm 1st}$ and $\lambda_{\rm SSB}$.

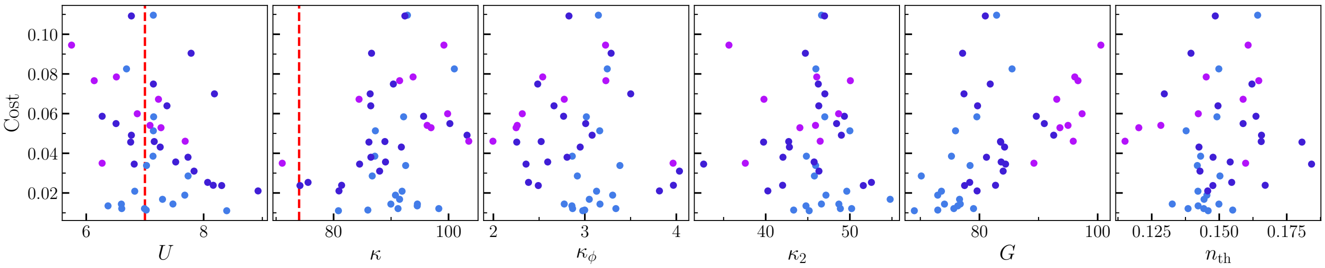

Pump (A.u.)	L	U [kHz]	κ [kHz]	κ_ϕ [KHz]	κ_2 [Hz]	G [KHz]	$n_{\rm th}$	Cost Range
0.85	1	6.3 – 8.3	80.8 – 98.3	2.7 – 3.3	43 – 55	68 – 79	0.13 – 0.15	0.011 – 0.029
0.95	1.12	6.7 – 8.9	74 – 103	2.2 – 4.0	32 – 52	77 – 93	0.14 – 0.18	0.021–0.046
1.05	1.29	5.8 – 7.6	71 – 103	2.0 – 4.0	35 – 50	89 – 100	0.11 – 0.16	0.034 – 0.094

Supplementary Figure 17. Distribution of the obtained parameter vs their cost function, and table summarizing the best estimated parameters obtained through simulated annealing. The red vertical lines indicate the experimentally obtained estimation.

A. Testing the validity of the model

First, we aim at verifying the validity of the proposed model in (48), in particular in determining the Liouvillian gaps. To do that, we see if the result of our estimation retrieves a value of U and κ that is comparable with the experimentally measured ones.

First, we introduce the cost function. Let $\vec{p} = [U, \kappa, \kappa_\phi, \kappa_2, G, n]$ be the set of parameters. We compute, for the same set of frequencies experimentally measured $\{\omega_1 \dots \omega_n\}$, the photon number $\vec{n}^{\rm th} = \{n(\omega_1), \dots n(\omega_n)\}$. We then select 6 points around the minimum of the first-order gap, and define $\vec{\lambda}^{\rm exp}_{\rm 1st}$, and numerically obtain the Liouvillian gap $\vec{\lambda}^{\rm th}_{\rm 1st}$ at the corresponding . We do the same for the SSB gap $\vec{\lambda}^{\rm exp}_{\rm SSB}$, and obtain the corresponding numerical data $\vec{\lambda}^{\rm th}_{\rm SSB}$. We then introduce the cost function

$$C = d(\vec{n}^{\rm exp}, \vec{n}^{\rm th}) + 4d(\vec{\lambda}^{\rm exp}_{\rm 1st}, \vec{\lambda}^{\rm th}_{\rm 1st}) + 6d(\vec{\lambda}^{\rm exp}_{\rm SSB}, \vec{\lambda}^{\rm th}_{\rm SSB}), \tag{49}$$

where d evaluates the distance between the theoretical result and the corresponding experimental one, once they have been normalized.

We then sort to a simulated annealing algorithm for probabilistic optimization, as we have to search for the optimal solution in a multi-dimensional space. The routine is

- Initialization: We set initially the parameters based on the experimental estimation and preliminary simulation of the remainder parameters.
- Set the temperature that controls the probability of accepting more costly solutions. Heuristically, we find a 50% rejection rate by setting the initial temperature to $T_0 = 0.1$.
- Compute the initial cost C.
- We then enter an interative loop where, for 20 iterations, we repeat
  - Update the temperature according to, in our case, an exponential schedule, reading $T = T_0 \times 0.93^{\rm iteration}$.
  - We extract a new set of random parameters, differing from the old one by a Gaussian function with variance 5% of the parameter.
  - Compute the cost function $C_{\rm new}$ for this new set of parameters.
  - If $C_{\rm new} < C$, accept the move. Otherwise, extract a random number r, and accept the move if $r < \exp[(C_{\rm new} - C)/T]$. In both cases, set $C = C_{\rm new}$. Otherwise, discard the move.

Following this strategy, we find the set of parameters indicated in Fig. 17. Importantly, the data agree with the experimental findings, and allow us to conclude that the model used is indeed predictive of the emergent physics.

B. Determination of the final parameter set

As we obtain consistent results through the various pump powers, we then assume a "one parameter-fit-all" strategy, fixing the values of the the Kerr nonlinearity U and total photon loss rate κ to the experimentally obtained value. We then re-run the simulated annealing algorithm, but this time compering the theoretical data with the experimental ones for all points at $\Delta/2\pi < 1$MHz. The result of this optimization are the parameters reported in the main text.

Supplementary Note 6. THEORY OF DISSIPATIVE PHASE TRANSITIONS

A. Open-system dynamics and Quantum Trajectories

The Lindblad master equation in (48) describes the state of an open quantum system at a time t via a density matrix ρ. Within a quantum trajectory approach, instead, the density matrix can be thought as a statistical mixture of pure states

$$\rho(t) = \lim_{N\to\infty} \frac{1}{N} \sum_{n=1}^{N} |\psi_n(t)\rangle \langle\psi_n(t)| \,, \tag{50}$$

where the evolution of the pure quantum states composing the mixture $\{|\psi_n(t)\rangle\}$ evolves according to a given stochastic protocol. The quantum expectation values can be obtained by averaging the over many of those states. When the number of trajectories N is large enough one recover the result of the Lindblad master equation with a statistical error that scales as $N^{-1/2}$. Below, we will use the following notation:

- $\langle\hat{o}\rangle(t) = \mathrm{Tr}\left[\rho(t)\hat{o}\right]$ indicates the average obtained either by the master equation or by an infinite number of trajectories.
- $\langle\hat{o}\rangle_\Psi(t) = \langle\Psi(t)|\hat{o}|\Psi(t)\rangle$ indicates the expectation value of a single quantum trajectory $|\Psi(t)\rangle$.
- Given a generic function f, we define the average over (ideally infinitely many) trajectories as $\overline{f(\langle\hat{o}\rangle_\Psi(t))} = \lim_{N\to\infty} \frac{1}{N}\sum_{n=1}^{N} f(\langle\hat{o}\rangle_{\Psi_n}(t))$.

1. *Ergodicity*

In many experimental data reported above, instead of performing a measure over many quantum trajectories, we rather measured an extremely long one, and then averaged over such measures. This property is nothing but an *ergodicity* of the quantum trajectory, that explores the entirety of the probability space of the steady state.

The proof of the ergodicity of a single quantum trajectory (in system with a single zero eigenvalue) goes as follows:

- Consider an initial condition $|\Psi(t=0)\rangle$ and evolve it to the long time limit, $|\Psi(t=T)\rangle$ with $T \gg 1/\lambda_{\rm 1st}$, $T \gg 1/\lambda_{\rm 2nd}$. At this point in time, the quantum trajectory will have lost any memory of the initial condition. Indeed, of we were to average over many quantum trajectories, we would recover $\rho_{\rm ss}$. In other words, $|\Psi(T)\rangle$ is one of the states of $\rho_{\rm ss}$.
- We reinitialise the system, by considering a set of trajectories $|\Phi_n(t=0)\rangle = |\Psi(t=T)\rangle$, that is the initial state of our second simulation is the final state of the first one.
- Since the average over many $|\Phi_n(t=T)\rangle$ must recover $\rho_{\rm ss}$, we deduce that each $|\Phi_n(t=0)\rangle$ can evolve with a certain probability towards one of the states composing $\rho_{\rm ss}$.
- Given that the time evolution of the quantum trajectory is Markovian, every one of the trajectories $|\Phi_n(t=0)\rangle$ is a legitimate evolution for $|\Psi(t=T)\rangle$.
- Since this line of reasoning can be extended to all the states $|\Psi(t>T)\rangle$, we conclude that a single quantum trajectory must explore all the states of $\rho_{\rm ss}$, and the average for long times must exactly recover the average over many trajectories.

B. The Liouvillian spectrum and phase transitions

The steady-state of a system can display a nonanalitical behavior as a function of one parameter. In this case, we say that a phase transition is taking place. Here, we briefly recall the spectral properties of the Liouvillian, and how

they can signal the emergence of phase transitions [21]. The interested reader may find a more detailed discussion of the peculiar properties of DPTs also in [20, 22–31].

Given any Liouvillian $\mathcal{L}$, we can introduce its eigenvalues λ_i and eigenmatrices ρ_i, defined via the relation

$$\mathcal{L}\rho_i = \lambda_i \rho_i. \tag{51}$$

It can be proved [32, 33] that $\mathrm{Re}\{\lambda_i\} \leq 0, \forall i$. For convenience, we sort the eigenvalues in such a way that $|\mathrm{Re}\{\lambda_0\}| < |\mathrm{Re}\{\lambda_1\}| < \ldots < |\mathrm{Re}\{\lambda_n\}|$. Usually, there exists a unique steady state density matrix $\rho_{\mathrm{ss}} \propto \rho_0$ such that $\mathcal{L}\rho_{\mathrm{ss}} = 0$, i.e., the steady state does not evolve anymore under the action of the Liouvillian superoperator. In this configuration, the real part of the eigenvalues is responsible for the relaxation towards the steady-state, while the complex values of λ_i describe oscillation processes in the dynamics. The eigenmatrix ρ_1 (the one associated to the smallest nonzero eigenvalue λ_1) describes the slowest relaxing state towards the steady state, and $\rho_{\mathrm{ss}} = \lim_{t\to+\infty} e^{-\mathcal{L}t}\rho(0)$.

Knowing the full Liouvillian spectrum, and aside from points where the Liouvillian is defective [34] one can immediately write the dynamics of any density matrix as [35]

$$\rho(t) = \rho_{\mathrm{ss}} + \sum_j c_j e^{-\lambda_j t}\rho_j \tag{52}$$

where the coefficient c_j can be determined using the left eigenoperators of the Liouvillian.

Consider now a system which, in a certain region of the space parameters, admits a unique steady state. In the thermodynamic limit $N \to +\infty$, a transition between two different phases is characterized by the nonanalytical behavior of some ζ-independent observable $\hat{o}$ when the parameter ζ tends to the critical value ζ_c. Formally, we say that there is a phase transition of order M if

$$\lim_{\zeta\to\zeta_c}\left|\lim_{N\to+\infty}\frac{\partial^M}{\partial\zeta^M}\mathrm{Tr}\{\rho_{\mathrm{ss}}(\zeta,N)\hat{o}\}\right| = +\infty. \tag{53}$$

Roughly speaking, this phase transition takes place in the thermodynamic limit when some eigenvalues pass from being nonzero to become exactly zero, both in its real and imaginary parts, as a function of the parameter ζ.

In finite-size systems, phase transitions cannot be observed. Nevertheless, the study of the Liouvillian eigenvalues provides much useful information about the scaling and nature of the transition [36].

1. *Second order phase transition*

A weak symmetry of an open quantum system is described by the presence of a superoperator $\mathcal{U} = \hat{V} \bullet \hat{V}^{-1}$ [37], such that

$$\mathcal{U}^{-1}\mathcal{L}\mathcal{U} = \mathcal{L}, \tag{54}$$

or, equivalently, $[\mathcal{L},\mathcal{U}] = 0$. The symbol $\bullet$ is a placeholder, and $\mathcal{U}\rho = (\hat{V}\bullet\hat{V}^{-1})\rho = \hat{V}\rho\hat{V}^{-1}$ The presence of a symmetry in the system fixes many properties of the system. Indeed, if ρ_{ss} is the only eigenmatrix with zero eigenvalue of $\mathcal{L}$ (unique steady state) before the transition, one can prove that it must also be an eigenmatrix of $\mathcal{U}$, and $\mathcal{U}\rho_{\mathrm{ss}} = \rho_{\mathrm{ss}}$ (ρ_{ss} is a symmetric state).

Let us consider now the Z_2 symmetry of the model in (48). In this case, the symmetry superoperator is $\mathcal{U} = \hat{\Pi} \bullet \hat{\Pi}$ where $\hat{\Pi} = \exp\{i\pi\hat{a}^\dagger\hat{a}\}$ is the parity operator. In this case, the phase transition is associated to one eigenvalue λ_{SSB} becoming and remaining zero in a whole region. The corresponding states ρ_{SSB}, allow to construct the symmetry-breaking metastable state. Indeed, one can construct $\rho^{\pm}_{\mathrm{SSB}} = \rho_{\mathrm{ss}} \pm \rho_{\mathrm{SSB}}$, which are well-defined density matrices that decay at a rate λ_{SSB}, and $\mathcal{U}\rho^{\pm}_{\mathrm{SSB}} = \rho^{\mp}_{\mathrm{SSB}}$ (they are not symmetric).

2. *First-order dissipative phase transition and the Liouvillian spectrum*

For a first-order phase transition, ρ_{ss} must be discontinuous and the transition is signalled by λ_{1st} In Ref. [21], it was proved that $\rho_{\mathrm{1st}} \propto \rho^{+}_{\mathrm{1st}} - \rho^{-}_{\mathrm{1st}}$, ρ^{+}_{1st} (ρ^{-}_{1st}) being the density matrix just before (after) the phase transition. Moreover, at the critical point $\rho_{\mathrm{ss}} \propto \rho^{+}_{\mathrm{1st}} + \rho^{-}_{\mathrm{1st}}$. Let us note that this equation has a clear physical interpretation: at the critical point, for a finite-size system, the steady state is the equiprobable mixture of the two phases, which are encoded in the spectral decomposition of ρ_{1st}. In a region at the left (right) of the critical point, ρ^{+}_{1st} (ρ^{-}_{1st}) are metastable [38, 39]. This means that if the system is initialized in one of these two states it will remain stuck, for a time proportional to $1/\lambda_{\mathrm{1st}}$, before reaching the steady-state [35]. This can gives rise to hysterical behaviour, typical of first-order phase transitions [40].

C. Extracting the Liouvillian gap from symmetry breaking trajectories

We explore here the relation between the dynamics of a single quantum trajectory and the Liouvillian eigenvalues, and demonstrate the equation

$$\begin{aligned} C_{\rm ss}(t) &= \lim_{\tau,\, T\to\infty} \frac{1}{T}\int_{\tau}^{\tau+T} \frac{I(\tau')I(t+\tau')}{I^2(\tau')} d\tau' \\ &\simeq \exp\{-\lambda_{\rm SSB} t\} \end{aligned} \tag{55}$$

.

Let us consider a two-point correlation function for quantum trajectories, i.e.,

$$\overline{\langle \hat{o}\rangle_\Psi (t)\, \langle \hat{p}\rangle_\Psi (t')} = \lim_{N\to\infty} \sum_{n=1}^{N} \frac{\langle \hat{o}\rangle_{\Psi_n} (t)\, \langle \hat{p}\rangle_{\Psi_n} (t')}{N}. \tag{56}$$

To understand the meaning of these object, we need to introduce the idea of the probability space of the trajectories [36, 41]. In this formalism, the density matrix initial pure state can be formally written as an integral over the space of trajectories $\mathcal{H}$ as

$$\rho(t) = e^{-\mathcal{L}t}\, |\Psi(0)\rangle\langle\Psi(0)| = \int_{\mathcal{H}} {\rm d}\Psi(t)\; p\big[\Psi(t)|\Psi(0)\big]\hat{\rho}_\Psi(t), \tag{57}$$

where $p\big[\Psi(t)|\Psi(0)\big]$ indicates the conditional probability of obtaining $|\Psi(t)\rangle$ given the initial condition $|\Psi(0)\rangle$, and $\hat{\rho}_\Psi(t) = |\Psi(t)\rangle\,\langle\Psi(t)|$. Since the steady state is independent of the initial condition, we have

$$\rho_{\rm ss} = \int_{\mathcal{H}} {\rm d}\Psi\; p_{\rm ss}\big[\Psi\big]\hat{\rho}_\Psi. \tag{58}$$

In this notation, we have

$$\begin{aligned} \overline{\langle \hat{o}\rangle_\Psi (t)\, \langle \hat{p}\rangle_\Psi (t')} &= \iint_{\mathcal{H}} {\rm d}\Psi(t)\; {\rm d}\Psi(t')\; p\big[\Psi(t)|\Psi(0), \Psi(t')|\Psi(0)\big]\, \langle \hat{o}\rangle_\Psi (t)\, \langle \hat{p}\rangle_\Psi (t') \\ &= \int_{\mathcal{H}} {\rm d}\Psi(t) p\big[\Psi(t)|\Psi(0)\big]\, \langle \hat{o}\rangle_\Psi (t) \int_{\mathcal{H}} p\big[\Psi(t')|\Psi(t)\big]\, \langle \hat{p}\rangle_\Psi (t'). \end{aligned} \tag{59}$$

where $p\big[\Psi(t), \Psi(t')|\Psi(0)\big]$ is the joint probability of having $|\Psi(t)\rangle$ and $|\Psi(t')\rangle$ given the initial condition $|\Psi(0)\rangle$, and we used the fact that

$$p\big[\Psi(t)|\Psi(0), \Psi(t')|\Psi(0)\big] = p\big[\Psi(t)|\Psi(0)\big]p\big[\Psi(t')|\Psi(t)\big]. \tag{60}$$

The latter follows from the fact that the conditional probability of $\Psi(t')$ depends only on the intermediate state $\Psi(t)$, but not on the previously visited state such as $\Psi(0)$, since a quantum trajectory is a Markovian process.

The second term of Eq. (59) is now identical to Eq. (57), and thus we can re-write it as

$$\int_{\mathcal{H}} p\big[\Psi(t')|\Psi(t)\big]\, \langle \hat{p}\rangle_\Psi (t') = {\rm Tr}\left[\hat{p} \int_{\mathcal{H}} p\big[\Psi(t')|\Psi(t)\big]\, |\Psi(t')\rangle\, \langle\Psi(t')|\right] = {\rm Tr}\left[\hat{p}\left(e^{-\mathcal{L}(t'-t)}\hat{\rho}_\Psi(t)\right).\right] \tag{61}$$

Passing back to the definition in terms of quantum trajectories, we have

$$\overline{\langle \hat{o}\rangle_\Psi (t)\, \langle \hat{p}\rangle_\Psi (t')} = \lim_{N\to\infty} \sum_{n=1}^{N} \frac{{\rm Tr}\left[\hat{o}\hat{\rho}^n_\Psi(t)\right] {\rm Tr}\left[\hat{p}\left(e^{\mathcal{L}(t'-t)}\hat{\rho}^n_\Psi(t)\right)\right]}{N}. \tag{62}$$

One can now use Eq. (52) and write

$$\hat{\rho}^n_\Psi = \rho_{\rm ss} + \sum_j c^n_j(t)\, \rho_j \tag{63}$$

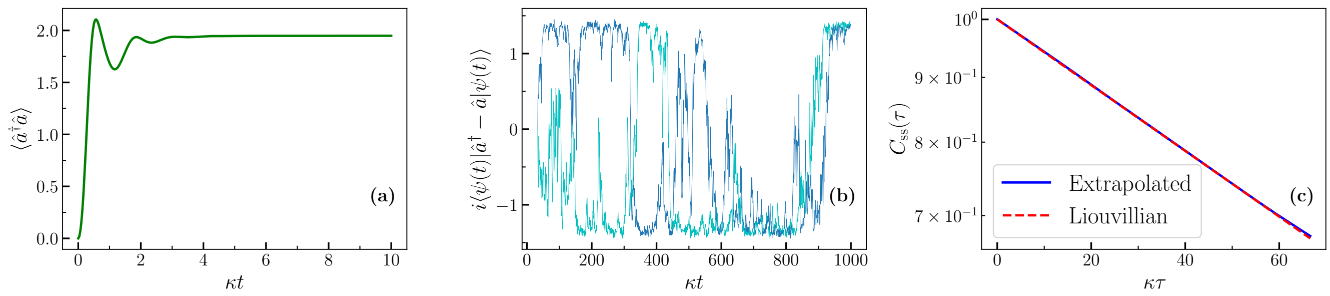

Supplementary Figure 18. **Correlation function and Liouvillian gap**. (a) Photon number evolution according to the Lindblad master equation in (48). (b) Single quantum trajectories performing heterodyne measurement [42]. (c) As a function of time, the correlation function $C_{\rm ss}(t)$, and $\exp\{\lambda_{\rm SSB} t\}$, the latter having being obtained by numerical diagonalization of the Liouvillian. Parameters: $\Delta = 0$, $U/\kappa = 1$, $G/\kappa = 2$, $\kappa_\phi/\kappa = 0$, $\kappa_2/\kappa = 0.1$, and $n_{\rm th} = 0$.

Using the spectral decomposition of the Liouvillian we can rewrite as $\hat{\rho}^n_\Psi(t) = \rho_{\rm ss} + \sum c^n_i(t)\rho_i$, and finally

$$\overline{\langle \hat{o} \rangle_\Psi (t) \, \langle \hat{p} \rangle_\Psi (t')} = \lim_{N\to\infty} \frac{1}{N} \sum_n {\rm Tr}\left[\hat{o}\hat{\rho}^n_\Psi(t)\right] {\rm Tr}\left[\hat{p}\left(\rho_{\rm ss} + \sum_i e^{-\lambda_i(t'-t)} c^n_i(t)\rho_i\right)\right]. \tag{64}$$

We send now $t \to \infty$, so that the coefficient $c^n_i(t) \to c^n_{i,{\rm ss}}$ are time-independent. We then call the time difference $\Delta t = t' - t$, and we suppose that Δt is large, so we can neglect all but the eigenvalue λ_1 the closest to zero in real part. We obtain

$$\begin{aligned}
\overline{\langle \hat{o} \rangle^{\rm ss}_\Psi \, \langle \hat{p} \rangle_\Psi (\Delta t)} &\simeq \lim_{N\to\infty} \frac{1}{N} \sum_n {\rm Tr}\left[\hat{o}\hat{\rho}^n_\Psi(t)\right] {\rm Tr}\left[\hat{p}\left(\rho_{\rm ss} + e^{-\lambda_1 \Delta t} c^n_{i,{\rm ss}}\rho_i\right)\right] \\
&= \lim_{N\to\infty} \frac{1}{N} \sum_n {\rm Tr}\left[\hat{o}\hat{\rho}^n_\Psi(t)\right] \left\{\langle \hat{p} \rangle_{\rm ss} + {\rm Tr}\left[\hat{p} e^{-\lambda_1 \Delta t} c^n_i \rho_i\right]\right\} \\
&= \langle \hat{o} \rangle_{\rm ss} \langle \hat{p} \rangle_{\rm ss} + \lim_{N\to\infty} \frac{e^{-\lambda_1 \Delta t}}{N} \sum_n {\rm Tr}\left[\hat{o}\hat{\rho}^n_\Psi\right] {\rm Tr}\left[\hat{p} c^n_1 \rho_1\right] = \langle \hat{o} \rangle_{\rm ss} \langle \hat{p} \rangle_{\rm ss} + e^{-\lambda_1 \Delta t}\mathcal{R},
\end{aligned} \tag{65}$$

where $\mathcal{R} = \sum_n {\rm Tr}\left[\hat{o}\hat{\rho}^n_\Psi\right] {\rm Tr}\left[\hat{p} c^n_1 \rho_1\right]$. The correlation function in Eq. (14) is a re-normalization of Eq. (65). Hence, we conclude that, by studying the evolution of a single trajectory level, we can access the value of the Liouvillian gap. This is numerically demonstrated in Fig. 18.

SUPPLEMENTARY REFERENCES

[1] L. Gravina, F. Minganti, and V. Savona, Critical schrödinger cat qubit, PRX Quantum **4**, 020337 (2023).
[2] D. Ruiz, R. Gautier, J. Guillaud, and M. Mirrahimi, Two-photon driven Kerr quantum oscillator with multiple spectral degeneracies, Phys. Rev. A **107**, 042407 (2023).
[3] J. Venkatraman, R. G. Cortinas, N. E. Frattini, X. Xiao, and M. H. Devoret, A driven quantum superconducting circuit with multiple tunable degeneracies (2022), arXiv:2211.04605 [quant-ph].
[4] M. Dykman, *Fluctuating nonlinear oscillators: from nanomechanics to quantum superconducting circuits* (Oxford University Press, 2012).
[5] E. P. Menzel, R. Di Candia, F. Deppe, P. Eder, L. Zhong, M. Ihmig, M. Haeberlein, A. Baust, E. Hoffmann, D. Ballester, K. Inomata, T. Yamamoto, Y. Nakamura, E. Solano, A. Marx, and R. Gross, Path entanglement of continuous-variable quantum microwaves, Phys. Rev. Lett. **109**, 250502 (2012).
[6] C. Eichler, D. Bozyigit, and A. Wallraff, Characterizing quantum microwave radiation and its entanglement with superconducting qubits using linear detectors, Phys. Rev. A **86**, 032106 (2012).
[7] R. Di Candia, E. Menzel, L. Zhong, F. Deppe, A. Marx, R. Gross, and E. Solano, Dual-path methods for propagating quantum microwaves, New J. Phys. **16**, 015001 (2014).
[8] M. Wallquist, V. S. Shumeiko, and G. Wendin, Selective coupling of superconducting charge qubits mediated by a tunable stripline cavity, Phys. Rev. B **74**, 224506 (2006).
[9] C. Eichler and A. Wallraff, Controlling the dynamic range of a Josephson parametric amplifier, EPJ Quantum Technology **1**, 2 (2014).
[10] W. Wustmann and V. Shumeiko, Parametric resonance in tunable superconducting cavities, Phys. Rev. B **87**, 184501 (2013).
[11] P. Krantz, Y. Reshitnyk, W. Wustmann, J. Bylander, S. Gustavsseon, W. D. Oliver, T. Duty, V. Shumeiko, and P. Delsing, Investigation of nonlinear effects in Josephson parametric oscillators used in circuit quantum electrodynamics, New J. Phys. **15**, 105002 (2013).
[12] Q.-M. Chen, M. Partanen, F. Fesquet, K. E. Honasoge, F. Kronowetter, Y. Nojiri, M. Renger, K. G. Fedorov, A. Marx, F. Deppe, and R. Gross, Scattering coefficients of superconducting microwave resonators. II. System-bath approach, Phys. Rev. B **106**, 214506 (2022).
[13] A. A. Clerk, M. H. Devoret, S. M. Girvin, F. Marquardt, and R. J. Schoelkopf, Introduction to quantum noise, measurement, and amplification, Rev. Mod. Phys. **82**, 1155 (2010).
[14] N. Bartolo, F. Minganti, W. Casteels, and C. Ciuti, Exact steady state of a Kerr resonator with one- and two-photon driving and dissipation: Controllable wigner-function multimodality and dissipative phase transitions, Phys. Rev. A **94**, 033841 (2016).
[15] W. Casteels, F. Storme, A. Le Boité, and C. Ciuti, Power laws in the dynamic hysteresis of quantum nonlinear photonic resonators, Phys. Rev. A **93**, 033824 (2016).
[16] A. Anferov, A. Suleymanzade, A. Oriani, J. Simon, and D. I. Schuster, Millimeter-wave four-wave mixing via kinetic inductance for quantum devices, Phys. Rev. Appl. **13**, 024056 (2020).
[17] S. M. Girvin, Circuit QED: superconducting qubits coupled to microwave photons, in *Quantum Machines: Measurement and Control of Engineered Quantum Systems: Lecture Notes of the Les Houches Summer School: Volume 96, July 2011* (Oxford University Press, 2014).
[18] S. Probst, F. B. Song, P. A. Bushev, A. V. Ustinov, and M. Weides, Efficient and robust analysis of complex scattering data under noise in microwave resonators, Review of Scientific Instruments **86**, 024706 (2015).
[19] M. S. Khalil, M. J. A. Stoutimore, F. C. Wellstood, and K. D. Osborn, An analysis method for asymmetric resonator transmission applied to superconducting devices, J. Appl. Phys. **111**, 054510 (2012).
[20] F. Minganti, V. Savona, and A. Biella, Dissipative phase transitions in n-photon driven quantum nonlinear resonators (2023), arXiv:2303.03355 [quant-ph].
[21] F. Minganti, A. Biella, N. Bartolo, and C. Ciuti, Spectral theory of liouvillians for dissipative phase transitions, Phys. Rev. A **98**, 042118 (2018).
[22] F. Minganti, I. I. Arkhipov, A. Miranowicz, and F. Nori, Liouvillian spectral collapse in the Scully-Lamb laser model, Phys. Rev. Res. **3**, 043197 (2021).
[23] F. Minganti, I. I. Arkhipov, A. Miranowicz, and F. Nori, Continuous dissipative phase transitions with or without symmetry breaking, New J. Phys. **23**, 122001 (2021).
[24] E. M. Kessler, G. Giedke, A. Imamoğlu, S. F. Yelin, M. D. Lukin, and J. I. Cirac, Dissipative phase transition in a central spin system, Phys. Rev. A **86**, 012116 (2012).
[25] H. J. Carmichael, Breakdown of photon blockade: A dissipative quantum phase transition in zero dimensions, Phys. Rev. X **5**, 031028 (2015).
[26] S. Lieu, R. Belyansky, J. T. Young, R. Lundgren, V. V. Albert, and A. V. Gorshkov, Symmetry breaking and error correction in open quantum systems, Phys. Rev. Lett. **125**, 240405 (2020).
[27] M. Soriente, T. L. Heugel, K. Omiya, R. Chitra, and O. Zilberberg, Distinctive class of dissipation-induced phase transitions and their universal characteristics, Phys. Rev. Res. **3**, 023100 (2021).
[28] M. Soriente, T. Donner, R. Chitra, and O. Zilberberg, Dissipation-induced anomalous multicritical phenomena, Phys. Rev. Lett. **120**, 183603 (2018).
[29] T. E. Lee, S. Gopalakrishnan, and M. D. Lukin, Unconventional magnetism via optical pumping of interacting spin systems,

Phys. Rev. Lett. **110**, 257204 (2013).
[30] J. Huber, P. Kirton, and P. Rabl, Nonequilibrium magnetic phases in spin lattices with gain and loss, Phys. Rev. A **102**, 012219 (2020).
[31] M. Biondi, G. Blatter, H. E. Türeci, and S. Schmidt, Nonequilibrium gas-liquid transition in the driven-dissipative photonic lattice, Phys. Rev. A **96**, 043809 (2017).
[32] H. Breuer and F. Petruccione, *The Theory of Open Quantum Systems* (Oxford University Press, Oxford, 2007).
[33] Á. Rivas and S. F. Huelga, *Open Quantum Systems: An Introduction* (Springer, Berlin, 2011).
[34] F. Minganti, A. Miranowicz, R. W. Chhajlany, and F. Nori, Quantum exceptional points of non-hermitian hamiltonians and liouvillians: The effects of quantum jumps, Phys. Rev. A **100**, 062131 (2019).
[35] K. Macieszczak, M. Guţă, I. Lesanovsky, and J. P. Garrahan, Towards a theory of metastability in open quantum dynamics, Phys. Rev. Lett. **116**, 240404 (2016).
[36] F. Vicentini, F. Minganti, R. Rota, G. Orso, and C. Ciuti, Critical slowing down in driven-dissipative Bose-Hubbard lattices, Phys. Rev. A **97**, 013853 (2018).
[37] B. Baumgartner and N. Heide, Analysis of quantum semigroups with GKS-Lindblad generators: II. general, J. Phys. A: Math. Theor. **41**, 395303 (2008).
[38] H. Landa, M. Schiró, and G. Misguich, Multistability of driven-dissipative quantum spins, Phys. Rev. Lett. **124**, 043601 (2020).
[39] H. Landa, M. Schiró, and G. Misguich, Correlation-induced steady states and limit cycles in driven dissipative quantum systems, Phys. Rev. B **102**, 064301 (2020).
[40] S. R. K. Rodriguez, W. Casteels, F. Storme, N. Carlon Zambon, I. Sagnes, L. Le Gratiet, E. Galopin, A. Lemaître, A. Amo, C. Ciuti, and J. Bloch, Probing a dissipative phase transition via dynamical optical hysteresis, Phys. Rev. Lett. **118**, 247402 (2017).
[41] F. Vicentini, F. Minganti, A. Biella, G. Orso, and C. Ciuti, Optimal stochastic unraveling of disordered open quantum systems: Application to driven-dissipative photonic lattices, Phys. Rev. A **99**, 032115 (2019).
[42] H. Wiseman and G. Milburn, *Quantum Measurement and Control* (Cambridge University Press, Cambridge, 2010).
